# Petroleum hydrocarbon rich oil refinery sludge of North-East India harbours anaerobic, fermentative, sulfate-reducing, syntrophic and methanogenic microbial populations

**DOI:** 10.1186/s12866-018-1275-8

**Published:** 2018-10-22

**Authors:** Ajoy Roy, Pinaki Sar, Jayeeta Sarkar, Avishek Dutta, Poulomi Sarkar, Abhishek Gupta, Balaram Mohapatra, Siddhartha Pal, Sufia K Kazy

**Affiliations:** 1 Department of Biotechnology, National Institute of Technology Durgapur, Durgapur, WB 713 209 India; 2 Department of Biotechnology, Indian Institute of Technology Kharagpur, Kharagpur, WB 721 302 India; 3 School of Bioscience, Indian Institute of Technology Kharagpur, Kharagpur, WB 721 302 India

**Keywords:** Refinery sludge, Microbial diversity, Total petroleum hydrocarbon, PICRUSt, Bioremediation

## Abstract

**Background:**

Sustainable management of voluminous and hazardous oily sludge produced by petroleum refineries remains a challenging problem worldwide. Characterization of microbial communities of petroleum contaminated sites has been considered as the essential prerequisite for implementation of suitable bioremediation strategies. Three petroleum refinery sludge samples from North Eastern India were analyzed using next-generation sequencing technology to explore the diversity and functional potential of inhabitant microorganisms and scope for their on-site bioremediation.

**Results:**

All sludge samples were hydrocarbon rich, anaerobic and reduced with sulfate as major anion and several heavy metals. High throughput sequencing of V3-16S rRNA genes from sludge metagenomes revealed dominance of strictly anaerobic, fermentative, thermophilic, sulfate-reducing bacteria affiliated to *Coprothermobacter*, *Fervidobacterium*, *Treponema*, *Syntrophus*, *Thermodesulfovibrio*, *Anaerolinea*, *Syntrophobacter, Anaerostipes, Anaerobaculum,* etc., which have been well known for hydrocarbon degradation. Relatively higher proportions of archaea were detected by qPCR. Archaeal 16S rRNA gene sequences showed presence of methanogenic *Methanobacterium*, *Methanosaeta*, *Thermoplasmatales*, etc. Detection of known hydrocarbon utilizing aerobic/facultative anaerobic (*Mycobacterium, Pseudomonas, Longilinea, Geobacter*, etc.), nitrate reducing (*Gordonia*, *Novosphigobium,* etc.) and nitrogen fixing (*Azovibrio, Rhodobacter*, etc.) bacteria suggested niche specific guilds with aerobic, facultative anaerobic and strict anaerobic populations. Phylogenetic Investigation of Communities by Reconstruction of Unobserved States (PICRUSt) predicted putative genetic repertoire of sludge microbiomes and their potential for hydrocarbon degradation; lipid-, nitrogen-, sulfur- and methane- metabolism. Methyl coenzyme M reductase A (*mcr*A) and dissimilatory sulfite reductase beta-subunit (*dsr*B) genes phylogeny confirmed methanogenic and sulfate-reducing activities within sludge environment endowed by hydrogenotrophic methanogens and sulfate-reducing *Deltaproteobacteria* and *Firmicutes* members.

**Conclusion:**

Refinery sludge microbiomes were comprised of hydrocarbon degrading, fermentative, sulfate-reducing, syntrophic, nitrogen fixing and methanogenic microorganisms, which were in accordance with the prevailing physicochemical nature of the samples. Analysis of functional biomarker genes ascertained the activities of methanogenic and sulfate-reducing organisms within sludge environment. Overall data provided better insights on microbial diversity and activity in oil contaminated environment, which could be exploited suitably for in situ bioremediation of refinery sludge.

**Electronic supplementary material:**

The online version of this article (10.1186/s12866-018-1275-8) contains supplementary material, which is available to authorized users.

## Background

Petroleum industries generate huge quantities of oily sludge containing various hydrocarbons and other recalcitrant compounds which may lead to severe environmental pollution due to its wide distribution, persistence and toxic nature [1, 2]. It has been estimated that around 8 × 10^4^ to 1 × 10^7^ tons of petroleum hydrocarbons will be globally released per year [3]. In India, more than 28,000 tons of oil containing sludge is generated annually by oil refineries [4]. Management and disposal of such hazardous waste remain a challenging problem worldwide. Over the years various physical and chemical methods have been developed for the treatment of oil-impacted sludge/environment. Nevertheless, eco-friendly, cost-effective and sustainable microbe-based in situ bioremediation of contaminated sites has gained considerable importance, as bioremediation technology is relying on the metabolic activity of the native microbial populations contributing in hydrocarbon mineralization through natural biogeochemical cycles [5, 6]. However, the diversity, distribution and activity of native microbial communities are determined by the prevailing environmental factors of the contaminated sites to thrive in such inhospitable conditions [7, 8]. Therefore, characterization of contaminated sites and inhabitant microbial populations have been considered as essential prerequisites for the development of bioremediation strategies as the success of bioremediation is very much influenced by the available physicochemical conditions and nutrient availability [9–11]. Bioremediation efficiency could be accelerated either by stimulating the metabolic activity of native microorganisms by nutrient amendments and/or by alterations in physicochemical conditions or by introducing more efficient microbes isolated from exogenous or endogenous sources [12–14].

Extensive culture-independent molecular investigations have revealed complex assemblages of diverse groups of aerobic and anaerobic microorganisms capable of various hydrocarbon degradation, nitrate−/sulfate−/metal-reduction, fermentation, syntrophism and methane metabolism in different oil-associated environments [10, 15–18]. Advanced meta-omics technologies have also demonstrated strong metabolic interactions within existing community owing to co-existence of diverse hydrocarbon-degrading microbial groups and catabolic genes related to aerobic and anaerobic degradation, nutrient metabolism and biogeochemical cycles in oil-associated environments [6, 19–25]. Several hydrocarbon metabolizing bacteria under the genera *Pseudomonas*, *Rhodococcus*, *Acinetobacter*, *Burkholderia*, *Sphingomonas*, *Alcanivorax, Marinobacter*, *Cycloclasticus,* etc., and their relevant catabolic genes have been well documented by previous investigators [10, 18–20, 22, 23, 26–28]. Recently, Hu et al., [29] revealed the potential roles of various candidate phyla like OP11, OP9, OD1, TA06, WS6 and SAR406 in the biogeochemical transformation of petroleum oils. Significant contributions of archaeal community in petroleum-contaminated environments have been established [30]. Ghosal et al., [18] have reported archaea as one of the six key members with hydrocarbon metabolizing properties along with *Alphaproteobacteria*, *Betaproteobacteria*, *Gammaproteobacteria*, *Actinomycetes* and *Firmicutes*. Many members of *Deltaproteobacteria* are known to be involved in anaerobic hydrocarbon degradation and their abundance often increased with petroleum hydrocarbon contamination [27, 31]. In recent years, high throughput sequencing technologies revealed the presence and the importance of less abundant rare microbial groups for maintaining ecosystem functions in diverse habitats. Obligate hydrocarbonoclastic bacteria are considered as “conditionally rare taxa” as these organisms showed huge abundance after drastic environmental perturbations due to oil spills [2]. Previous investigators have successfully used various functional biomarker genes such as alkane monooxygenase (*alk*B, which catalyses hydroxylation of alkanes), naphthalene dioxygenase (*ndo*, involved in hydroxylation of aromatic hydrocarbons), benzyl succinate synthase (*bss*A*,* anaerobic toluene degradation), methyl coenzyme M reductase A (*mcr*A, involved in the terminal step in anaerobic methane production) and dissimilatory sulfite reductase genes (*dsr*A and *dsr*B, involved in dissimilatory sulfite reduction and often associated with mineralization of organic compounds) to assess the catabolic potential of native microbial populations and the magnitude of contamination at the polluted sites [20, 25, 32–38]. Quantitative PCR (qPCR) based studies on such functional biomarkers have demonstrated the relationship between the abundance/expression of genes and biodegradation of contaminants [3, 26, 33]. Dombrowski et al., [39] proposed metabolic linkages amongst different functional groups, like fermentative members of the community which have substrate-level interdependencies with sulfur- and nitrogen-cycling microorganisms.

Extensive research has elucidated microbial communities from a variety of hydrocarbon contaminated environments including deep water horizon spill, oil contaminated soil or sediments, drill cuttings, production and injection water, etc. [3, 19–23, 26, 27, 37, 39, 40]. However, investigation on petroleum refinery waste sludge microbiome remained little explored. The present work was undertaken to explore the native microbial community composition and function in oil containing sludge of Guwahati and Digboi refineries, Assam, India, by adopting following approaches -i) 16S rRNA amplicon sequencing of sludge microbial communities using Illumina next-generation sequencing platform, ii) assessment of inherent microbial metabolic potentials using functional biomarker genes (*mcr*A and *dsr*B) through clone library and qPCR based studies and iii) predictive functional profiling of sludge microbial communities using PICRUSt. Our study provided deeper insights into the native microbial community composition and function in oily sludge, which might be exploited for in situ bioremediation of such petroleum refinery waste.

## Methods

### Sample collection, physicochemical analysis, and enumeration of microorganisms

Refinery waste sludge samples were collected in sterile screw-capped glass bottles (1 l capacity) using sterilized stainless steel scoops from storage tank/pit of Indian Oil Corporation Limited (IOCL) at Guwahati (26.18° N, 91.80° E and 26.14° N, E 91.73° E, designated as GR1 and GR3 respectively) and Digboi (27.39° N, 95.61° E designated as DB2), Assam, India and immediately stored in ice. Sludge samples were collected from nearly 30–50 cm below the top surface. Three to five subsamples were collected from each location and pooled together. The samples were immediately stored in sterile glass bottles (Schott Duran, Germany) at a temperature below 4 °C. All containers were sterile and nuclease-free. Physicochemical parameters including pH, temperature, oxidation-reduction potential (ORP), dissolved oxygen (DO) and conductivity of the samples were measured on site using an Orion Star 140 TM series meter (Thermo Electron Corporation, USA). The samples were stored at 4 °C after reaching laboratory.

Key anions (SO_4_^2−^, NO_3_^−^, NO_2_^−^, PO_4_^3−^, Cl^−^) were estimated by spectrophotometric and titrimetric methods (as appropriate) of American Public Health Association (APHA; methods 4500 and 2320B). Major metallic elements (Fe, Ni, Pb, Zn, Na, K, Cd) were estimated using inductively coupled plasma mass spectrophotometer (ICP-MS) (Varian 810 ICP-MS System, USA) following acid digestion (EPA protocol 3050B). Estimation of total petroleum hydrocarbon (TPH), as well as constituent hydrocarbons, was done by gravimetric method followed by gas chromatograph coupled with mass spectrometer (GC-MS) (Perkin Elmer Clarus 680, USA) analysis. Elite 5MS column (30 m × 0.25 mm id, film thickness 0.25 μm) was used along with helium as a carrier gas (flow rate 1 ml min^− 1^). The injector temperature was set at 260 °C. The oven temperature was initially set at 50 °C for 2 min and then increased to 60 °C at the rate of 2 °C min^− 1^ and maintained for 2 min, subsequently raised to 210 °C at 3 °C min^− 1^ and maintained for 2 min. Finally the oven temperature was raised to 270 °C at the rate of 10 °C min^−1^ and maintained for 7 min. The conditions for mass spectrophotometer operation were set as: ion source temperature, 200 °C; transfer line temperature was 280 °C; the mass range was 40–600 a.m.u (Atomic Mass Unit). Identification of the components was carried out by comparing the mass spectrum of the component to that of the mass spectral library from NIST 14 (National Institute of Standards and Technology, USA).

Total bacterial counts were ascertained using fluorescence microscopy following the protocol described by Kepner and Pratt [41]. Briefly, cells were dislodged from 0.1 g sample using 5 ml sterile sodium pyrophosphate (0.1% *w*/*v*), fixed with 4% paraformaldehyde and incubated at 4 °C for 4 h. Fixed cells were centrifuged (10,000 rpm for 5 min) and washed thrice in phosphate buffered saline (PBS) solution (pH 7.2). Staining was achieved using 0.1% (*w*/*v*) acridine orange (AO) to the resulting pellet and incubation in dark for 15 min. Excess of AO was washed with PBS and cells were visualized under Olympus CKX41 (Japan) inverted microscope at 1000X magnification with oil immersion. The software ImageJ (http://rsbweb.nih.gov/ij/) was used for quantification of cells. Enumeration of aerobic and anaerobic heterotrophic cell counts was done by resuspending 0.1 g of each sample in 9 ml sterile sodium pyrophosphate (0.1% *w/v*), followed by dilution of the extracted liquid up to 10^− 4^ using 0.9% (*w*/*v*) sterile saline. Finally the diluted samples were spread on Reasoner’s 2A (R2A), Minimal Salt Medium (MSM) agar and anaerobic agar (Himedia, India) [42]. For aerobic bacterial counts, R2A and MSM agar plates were incubated at 30 °C for 2 days. For anaerobic bacterial counts, anaerobic agar plates were incubated at 30 °C for 15 days in an anaerobic jar with the anaerobic gas pack (Himedia, India). Aerobic and anaerobic colonies were counted after 2 and 15 days, respectively.

### Community level physiological profiling (CLPP)

Functional potential of the microbial communities was investigated by community level physiological profiling (using Biolog ECO plate). Cells were dislodged from the samples using 1% (*w/v*) sodium pyrophosphate. 150 μl of the resulting cell suspension was inoculated in each well of the Biolog ECO plate and incubated at 30 °C. Along with the three waste sludge samples, a garden soil was taken as control (to appraise the possible effect of high TPH in substrate utilization). Utilization of substrates was measured by monitoring the optical density of each well at 590 nm, at an interval of every 12 h over 7 days. The average well color development (AWCD) was estimated according to the following equations:

$$ \mathrm{AWCD}=\sum \limits_{\mathrm{i}=0}^{31}{\mathrm{OD}}_{\mathrm{i}}/31 $$

### Metagenome extraction and sequencing of 16S rRNA genes

Total community DNA was extracted from each sample (250 mg) in triplicate using MoBioPowerSoil™ DNA extraction kit (MoBio, USA) according to the manufacturer’s instructions with slight modifications. Bead beating of the sample was done thrice for 5 min with an interval of 5 min. At the final step the DNA was eluted in nuclease free water. During elution of DNA from the spin column, after addition of water in the column it was incubated for half an hour which improved the DNA yield. The metagenomes were extracted at least 10 times from each sample and all the extractions were pooled together before amplicon sequencing. The DNA was quantified using Nanodrop spectrophotometer (Nano 2000 Thermo Fischer Scientific, USA) as well as Qubit fluorometer (Qubit 3.0 Fluorometer Applied Biosystem, USA) with A_260/280_ was in the range of 1.79–1.81. Microbial community in each sample was analyzed using Illumina based 16S rRNA gene (V3 region) amplicon sequencing. NGS services of Genotypic Technology Pvt. Ltd., Bangalore, India and SciGenome Labs, Chennai, India were used.

### Bioinformatic data analysis

Microbial diversity in terms of taxonomic groups as well as their abundance was determined using Quantitative Insights into Microbial Ecology (QIIME) version 1.9.1 [43] following Bartram et al. [44]. Paired-end raw reads obtained through Illumina sequencing of V3 region of 16S rRNA gene were merged into single end reads using FLASH (Fast Length Adjustment of Short Reads) with a minimum overlap of 8 bp and maximum mismatch density of 0.1 followed by conversion of the fastq file to their corresponding sequence and quality (.fna and .qual) files [45]. The sequence files were quality filtered using split_libraries.py. OTU (Operational Taxonomic Unit) picking and taxonomy assignment was done using pick_de_novo_otus.py with default parameters. Greengenes 13.8 database was used as a reference database for taxonomy assignment. Alpha diversity indices (Chao1, Shannon, Simpson, Good’s coverage and Observed species) as well as rarefaction curves were also estimated.

Venn diagrams were constructed to forecast the number of unique and shared entries at each taxonomic level viz., OTU, phyla, class, family and genus among the sludge samples using Venny 2.1 (http://bioinfogp.cnb.csic.es/tools/venny/). Weighted Pair Group Method with Arithmetic Mean (WPGMA) analysis of families (with cumulative abundance ≤0.2% abundance) based on Bray-Curtis dissimilarity index was done by MVSP (Multivariate Statistical Package).

Top 50 OTUs were selected on the basis of their cumulative abundances across all the samples and heat map was constructed on the basis of Pearson correlation using METAGENassist [46].

### Comparison of microbial communities of different petroleum hydrocarbon contaminated environments

Microbial community compositions (phylum level) of the test sludge samples were compared with fifteen other previously reported petroleum hydrocarbon contaminated environments. An UPGMA was performed among the samples using Euclidean similarity indices by using PAST3 software.

### Function prediction and metabolic pathway reconstruction

PICRUSt was used to predict the genomic repertoire of each community metagenome [47]. For PICRUSt analysis, the pick_closed_reference_otus.py command in QIIME was used with Greengenes version 13.5 as a reference database for OTU picking and the resulting OTU biom table was uploaded in the Galaxy server (https://huttenhower.sph.harvard.edu/galaxy/).This software assigns the functional features by comparing the identified 16S rRNA gene sequence with that of the closest match of the known genome sequence. Metagenome function was predicted with NSTI (Nearest Sequenced Taxon Index) values followed by the metabolic pathway reconstruction using KEGG (Kyoto Encyclopedia of Genes and Genomes) database. To calculate the closeness with known sequenced genomes, an indicator, nearest sequenced taxon index (NSTI), was calculated, wherein, a value close to 0 indicates high similarity to a closest sequenced taxon, whereas nearer to 1 indicates no significant similarity.

### Analysis of archaeal populations

To assess the archaeal diversity of the samples, archaeal specific 16S rRNA genes were PCR amplified from metagenome, cloned and clone libraries were analyzed through Sanger sequencing. Details of the primers used for amplification of archaebacterial 16S rRNA genes and PCR conditions are presented in Additional file 1: Table S1. Agarose gel purified PCR products of 16S rRNA genes were ligated into pTZ57R/T vector (Promega, USA) and transformed into *E. coli* DH10β following the manufacturer’s instructions. Randomly chosen (100–120) positive colonies per samples were picked up and analyzed for desired insert size. The cloned 16S rRNA gene fragments from each positive colony were re-amplified using vector specific primer M13F and M13R. Each library was subjected to ARDRA (Amplified Ribosomal DNA Restriction Analysis). The amplified products were digested with restriction endonucleases (*Hae*III and *Msp*I) in separate reactions. All digests were analyzed by 2.5% agarose gel electrophoresis [42]. ARDRA patterns were grouped visually and each group was referred as an OTU or ribotype. Plasmid DNA was isolated from selected clones of different OTUs using HiPura Plasmid DNA extraction kit (Himedia, India) according to the manufacturer’s instructions. Sequencing of the plasmid was done using M13R primer by Eurofins Genomics India Pvt. Ltd. For each sequence, closest sequences were retrieved from those available in public database by using the BLAST (NCBI) program (http://blast.ncbi.nlm.nih.gov/Blast.cgi) followed by initial classification using a web-based classifier program in ribosomal database project (RDP released 11 and with 95% of similarity) (http://rdp.cme.msu.edu/classifier/classifier.jsp). The phylogenetic tree was constructed using MEGA 5 with the neighbor-joining method [48].

### Real-time (qPCR) based quantification of bacterial- and archaeal 16S rRNA genes and *mcr*A and *dsr*B genes

Real-time q-PCR based on fluorescent dye SYBR green was used to quantify bacterial and archaeial 16S rRNA gene copies as well as the genes encoding methyl coenzyme M reductase (*mcr*A) and dissimilarity sulfite reduction (*dsr*B). Primer sequences used, amplification product sizes and annealing temperatures for respective genes are tabulated in Additional file 1: Table S1. Real-time PCR standard curves were prepared for absolute determination of copy number of each of the four genes. Each of the genes were PCR amplified from metagenome, cloned and positive clones containing correct inserts were used for plasmid extraction. Extracted and purified plasmids were re-sequenced for confirming the integrity of the cloned gene and then used as a standard for real-time PCR analysis.

Copy no. of each plasmid was calculated using the formulae:

$$ \mathrm{No}.\mathrm{of}\ \mathrm{copies}/\upmu \mathrm{L}=\frac{\left(\mathrm{Conc}.\mathrm{of}\ \mathrm{plasmid}\ \left[\mathrm{ng}/\upmu \mathrm{L}\right]\times \mathrm{Avogadro}\ \mathrm{No}.\right)}{\left(\mathrm{Size}\ \mathrm{of}\ \mathrm{plasmid}\times 660\times {10}^9\right)} $$

Standard curves were prepared for each gene with 10^− 2^–10^− 8^ dilutions of the original plasmid. Because lengths of the vector and target gene inserts were known, gene copy numbers could be estimated. Quantification was carried out on a QuantStudio 5 real-time PCR (Thermo-Fisher, USA) using QuantIt® SYBR green PCR master mix (Applied Biosystem, USA) following the manufacturer’s directions. Reaction mixtures (10 μL) contained 5 μL of 2X SYBR Green qPCR master mix, 0.5 μl for both the forward and reverse primer, pre-sequenced plasmids containing gene of interest and sample DNA as a template for standard curve preparation and gene quantification, respectively. Cycling conditions for real-time qPCR were as follows: hold for 10 min at 94 °C followed by 40 cycles of denaturing at 94 °C for 15 s, annealing at 55 °C for 15 s and extension at 72 °C for 15 s; followed by a melting curve from 55 °C to 95 °C (increment = 0.5 °C per 10 s). All samples were run in triplicate and melting curve of products was analyzed in order to monitor non-specific amplification or primer-dimers.

### Analysis of *mcr*A and *dsr*B genes

**Fig. 1:**
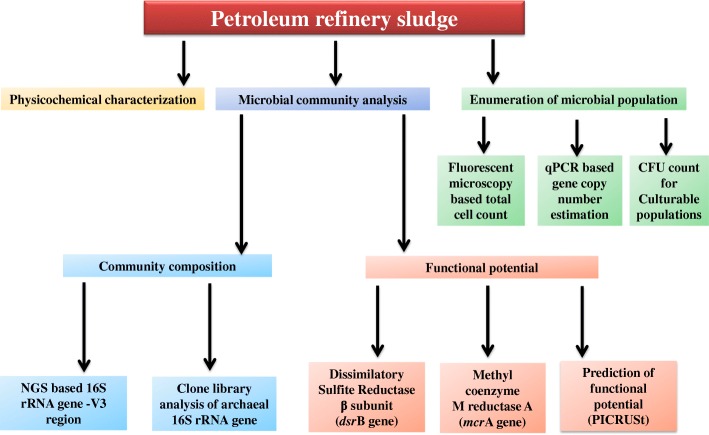
Detailed flow chart indicating the overall methodology followed in the present study

Nature and function of two important functional genes encoding the alpha subunit of methyl coenzyme M reductase (*mcr*A) and dissimilatory sulfite reductase subunit B (*dsr*B) were targeted to gain insight into the metabolic diversity in the petroleum-rich samples. *mcr*A gene was amplified using the specific primer pair ME1 and ME2. To elucidate the sulfate-reducing populations of the samples, clone library of the *dsr*B gene was constructed using gene-specific primers p2060F and 4R. Details of the primers used for each gene and PCR conditions are presented in Additional file 1: Table S1. Amplified PCR products were analyzed on 1% agarose gel; PCR products of *mcr*A and *dsr*B genes were gel purified (QIAquick gel extraction kit, Netherlands), resuspended in nuclease-free water and ligated into pTZ57R/T vector (Promega*,* USA). Ligated vectors were transformed into *E. coli* DH10β following the manufacturer’s instructions. Randomly chosen positive colonies were analyzed for desired insert size. Plasmid DNA was extracted from selected clones and sequencing of the inserted gene was done using M13R primer by Eurofins Genomics India Pvt. Ltd. Nucleotide sequences were translated using the ExPASy tools (http://www.expasy.org/tools/dna.html) and appropriate reading frame for each gene was selected. Protein homology of translated products was determined using BLASTP (NCBI). Phylogenetic trees were constructed using MEGA 5 with the neighbor-joining method [48]. Figure 1 showed a detailed flow chart indicating overall methodology followed in the present study.

## Results

### Physicochemical characteristics of sludge samples

Physicochemical parameters of the sludge samples were summarized in Table 1. All the three samples exhibited anoxic to strict anaerobic (DO: 0.01–0.66 mg l^− 1^), reducing condition with low conductivity. The temperature of the two samples GR1 and DB2 obtained from sludge storage pits were close to the local daytime temperature of the regions (35.2–35.6 °C), whereas GR3, collected from tank receiving the effluent from the refinery plant showed an elevated temperature of 44.9 °C. High TPH content with abundance of both aliphatic and aromatic hydrocarbons along with the presence of multiple heavy metals marked the characteristics of the three samples. GC-MS analysis revealed the presence of alkanes (C6-C40), cyclic- (C5, C6) and aromatic- (benzene, naphthalene, phenanthrene, etc.) hydrocarbons along with their substituents in the samples (Additional file 2: Table S2). Presence of several anions and heavy metals at varying concentrations was observed. Elevated sulfate concentration was detected in GR1 and DB2, while all three samples showed moderately high chloride concentrations with the highest amount in GR1. GR1 also showed relatively higher level of phosphate compared to others. Nitrate and nitrite were detected at lower concentrations in all the samples, although the level of nitrate was up to 6 times higher than nitrite. Among the heavy metals, iron was most abundant, followed by chromium, zinc, nickel and copper. Arsenic and lead were present at relatively low concentrations. It was noted that GR1 and DB2 shared commonality with respect to multiple parameters including ORP, TPH, heavy metals and anions than that of GR3.

### Quantification of microbial cells

Microbial abundance within the samples was quantified through total microscopic cell counts, enumeration of cultivable bacteria and real-time PCR (qPCR) based estimation of 16S rRNA gene copy numbers (Table 1). Total microscopic counts yielded 3.7–7.0 × 10^8^ cells g^− 1^ of GR1 and DB2 samples. For GR3 cell counts could not be obtained due to strong interference from its very high TPH content. Aerobic and anaerobic cultivable bacterial counts were nearly equal in GR3 and DB2 samples (~ 10^5^–10^6^ CFU g^− 1^), except in GR1 (anaerobic count was one order less). Microscopic and CFU data indicated that nearly 1% of the total cells present in these samples could be cultivable.

**Table 1:** Physicochemical and microbiological parameters of oily sludge samples

	GR1	GR3	DB2
Parameters			
Site	Guwahati Refinery, IOCL	Guwahati Refinery, IOCL	Digboi Refinery, IOCL
Geographical location	N 26^°^ 10.48′ E 091^°^ 48.00’	N 26^°^ 10.48′ E 091^°^ 48.00’	N 27^°^ 23.410′ E 095^°^ 36.665’
Nature of the sample	Oily sludge at waste disposal pit	Oily sludge at waste water lagoon	Oily sludge waste disposal pit
Physicochemical parameters			
Temperature (°C)^a^	35.2	44.9	35.6
pH ^a^	7.14	6.25	6.84
Dissolved Oxygen (mg/l) ^a^	0.66	0.19	0.01
ORP (mV) ^a^	− 157.7	−30	− 182
Conductivity (μS /cm) ^a^	0.3	1.4	0
Moisture content (%, *w*/w)	27.12	27.87	42.24
TPH (g/ kg)	143.8	400	140.2
Hydrocarbons detected (%)^b^			
Total Aliphatic Compounds	60	71	75
Total Aromatics	40	29	25
Ions (mg/kg)^c^			
Nitrate	18.24	7.87	13.63
Nitrite	2.97	< 2.0	9.07
Chloride	704.00	469.85	469.85
Sulfate	6109	222	6621
Phosphate	537.6	17	167
Ammonium	3.24	3.49	3920
Metals (mg/kg)^d^			
Sodium	3.13	2.23	3.56
Calcium	49.51	30.50	78.25
Chromium	45.39	8.07	48.38
Iron	150.47	302.97	101.13
Cobalt	1.34	2.39	2.53
Nickel	34.93	7.11	38.72
Copper	12.92	2.39	12.89
Zinc	34.70	131	68.14
Arsenic	2.24	1.27	2.22
Cadmium	0.06	0.16	0.05
Lead	2.68	4.02	2.62
Microbial Counts			
Total microbial counts using fluorescence microscopy (cells/g of sample)	(7.01 ± 0.87) × 10^8^	ND	(3.73 ± 0.68) × 10^8^
CFU/g of samples			
MSM	(1.65 ± 0.78) × 10^6^	0.2 × 10^6^	(5 ± 0.14) × 10^6^
R2A	(8 ± 1.41) × 10^5^	(6.5 ± 1.5) × 10^6^	(7.35 ± 0.21) × 10^6^
Anaerobic agar	(3 ± 0.14) × 10^5^	(2 ± 0.7) × 10^6^	(1.23 ± 0.14) × 10^6^
Gene of interest	Copy no./g sample		
Bacteria (16S rRNA gene)	4.46 × 10^9^	2.35 × 10^10^	3.13 × 10^9^
Archaea (16S rRNA gene)	2.54 × 10^8^	1.01 × 10^8^	1.04 × 10^8^
*mcr*A	1.75 × 10^6^	2.29 × 10^6^	1.66 × 10^6^
*dsr*B	2.22 × 10^8^	4.02 × 10^7^	3.48 × 10^8^

^a^Measured during sample collection using Orion star series multiparameter (Thermo Orion meter Beverly, USA)

^b^Analysed and Identified by gas chromatograph coupled with mass spectrometer (GC-MS) (Perkin Elmer, USA)

^c^Estimated by spectrophotometric and titrimetric methods of American Public Health Association

^d^Estimated by ICP-MS (inductively coupled mass spectrometery) (Varian Palo Alto CA USA) and /or atomic absorption spectroscopy (AAS) (Perkin Elmer MA, USA)

ND, Not detected

The concentration of metagenomic DNA extracted from the samples were in the range of 30-85 ng/μl. qPCR data indicated the presence of appreciable number of bacterial and archaeal 16S rRNA gene copies up to 2.35 × 10^10^ and 2.5 × 10^8^ per gram sample, respectively. Highest number of bacterial 16S rRNA gene copy was observed in GR3 and maximum archaeal 16S rRNA gene was present in GR1 (2.54 × 10^8^ copies/g). Assuming an average of 4.60 copies of 16S rRNA gene per bacterium and 1.71 per archaeal genome (https://rrndb.umms.med.umich.edu/), GR3 community was found to be hosted by 5.06 × 10^9^ cells g^− 1^ (5 × 10^9^ bacteria and 5.9 × 10^7^ archaebacteria), DB2 by 7.27 × 10^8^ cells g^− 1^(6.66 × 10^8^ bacteria and 6.12 × 10^7^ archaebacteria) and GR1 by 1.1 × 10^9^ cells g^− 1^ (9.49 × 10^8^ bacteria and 1.49 × 10^8^ archaebacteria). Estimated cell numbers obtained from microscopy and qPCR data corroborated well with each other, which ranged between 10^8^ and 10^9^ cells/g samples.

### Community level physiological profiling

**Fig. 2:**
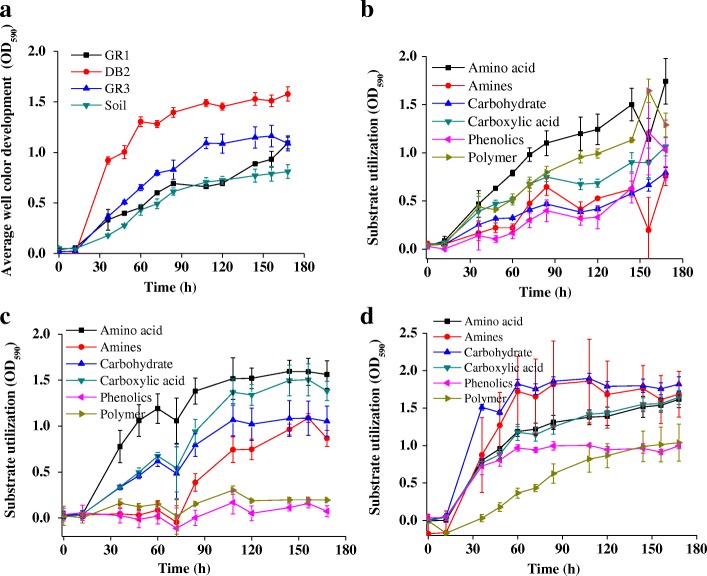
Community level physiological profiles of GR1, DB2 and GR3 sludge samples. Average well color development (AWCD) was plotted with time for the samples along with a non-polluted soil (Soil) (**a**). Color development was monitored till 180 h. Error bars represent the standard error of mean (*n* = 3). Utilization of seven categories of substrates by three sludge samples over time, GR1 (**b**), GR3 (**c**) and DB2 (**d**)

AWCD data indicated the rich physiological attributes of the refinery waste communities. Among the samples, DB2 and GR3 showed slightly more efficient utilization of the substrates provided in Biolog Eco Plate (Fig. 2a). Out of the six categories of substrates (amino acids, amines, carbohydrates, carboxylic acids, phenolics and polymers), amino acid was observed to be the most preferred in GR1 and GR3 while for DB2 it was carbohydrate followed by amines and amino acids (Fig. 2b, c, d). Overall, amino acids and amines were most and phenolics were least favored across the samples. Taking into consideration of the cell abundance, the observed metabolic richness highlighted the characteristic nature of these communities.

### Microbial community composition

The total number of sequence reads per sample varied between 435,940–627,638 and a total number of 25,923 OTUs (> 97% sequence similarity) could be obtained across the three samples (Table 2). Rarefaction analysis followed by Good’s coverage indicated satisfactory sampling for all the three libraries (Additional file 3: Figure S1). The alpha diversity indicators, viz., Chao1, equitability, Shannon’s and Simpson’s indices, were summarized and compared with other similar microbial habitats (Table 2 and Tables S3). Shannon diversity index for GR1 and DB2 were slightly higher than GR3, but all values were within the range as reported from several other petroleum production/injection wells, contaminated soil, etc. in recent years (Additional file 4: Table S3). At 97% similarity level, a minor fraction of the total OTUs (4.9%) was shared among the three samples, but accounted for ≥84% of the total sequences and leaving considerable fractions (16–41% of the OTUs) unique to each sample (Additional file 5: Figure S2). At taxonomic level, over 68% (41) phyla, 49% (135) families and 35% (140) genera were found to be common in the samples (Additional file 5: Figure S2). Highest numbers of taxa were found to be shared between DB2 and GR1 at both family and genus levels.

**Table 2:** Read and OTU distribution of the samples obtained through next generation sequencing (Illumina), diversity indices (calculated using QIIME workflow) and taxonomical distribution

	GR1	GR3	DB2
Parameters			
Number of reads	627,638	435,940	472,180
OTUs (97% identity)	7837	12,774	10,617
Estimated total OTUs (Chao1)	15,448.14	32,314.94	25,135.21
Shannon diversity index	6.148	4.443	6.369
Simpson index	0.9452	0.7081	0.95
Equitability	0.475	0.326	0.476
Goods coverage	0.9933	0.9819	0.9863
Archaeal taxa (% Reads)	0.03	0.0002	0.02
Bacterial taxa (% Reads)	95.41	99.14	97.07
Unclassified (% Reads)	4.56	0.86	2.91
Number of Archeal taxa detected			
Phylum	2	1	2
Class	3	1	5
Family	2	1	3
Genus	2	1	3
Genus ^e^	2	2	2
Genus common in 3 samples	1		
Number of Bacterial taxa detected			
Phylum	49	46	49
Class	118	96	116
Family	213	186	215
Genus	280	244	285
Genus common in 3 samples	139		

^e^Detected through clone library

### Beta diversity

**Fig. 3:**
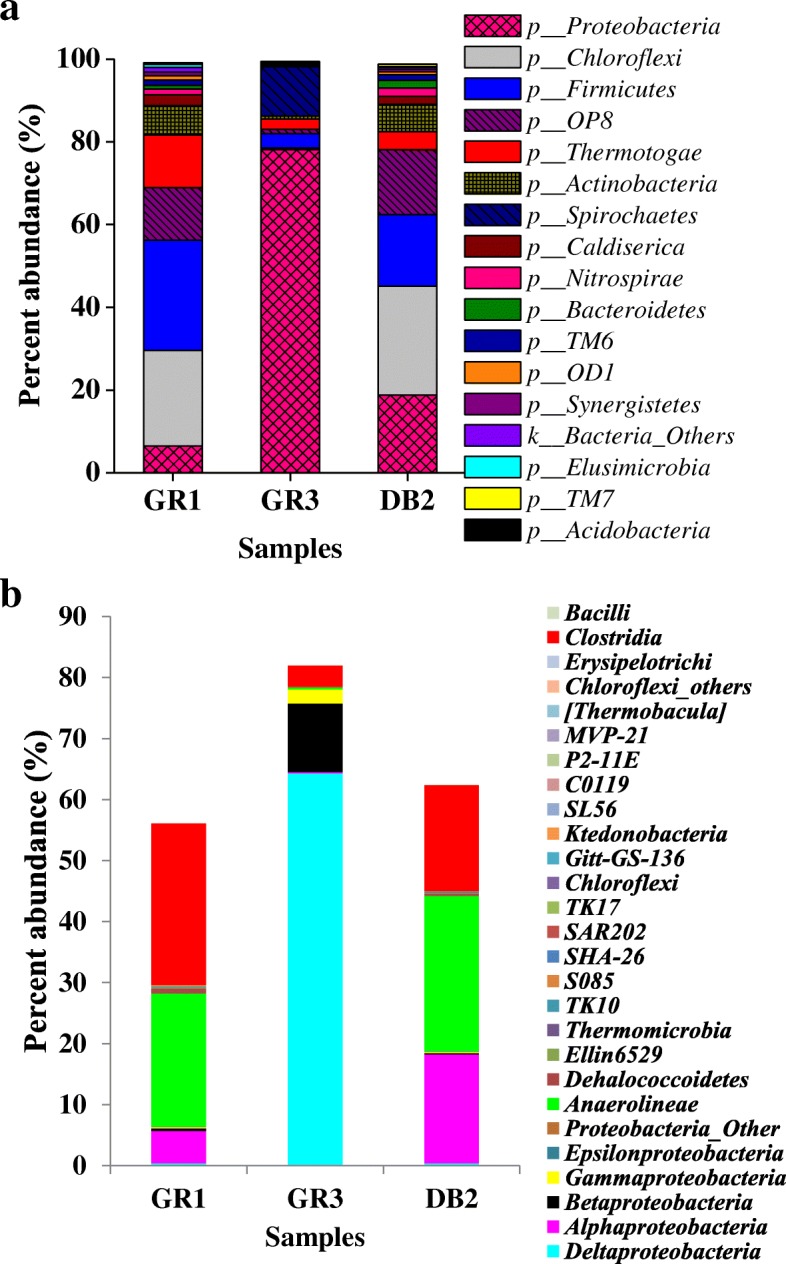
Composition of three microbial communities at phylum (**a**) and class (**b**) levels. Phyla with cumulative abundance ≥0.5% (across the three samples) were considered. Class level distribution was plotted for three major phyla *Proteobacteia*, *Chlroflexi* and *Firmicutes*

Distribution of bacterial phyla across the samples was delineated based on their relative abundance (Fig. 3a and Additional file 6: Figure S3). Members of *Proteobacteria, Chloroflexi, Firmicutes* and candidate division OP8 constituted major proportions (69–83%) in all the three communities. *Thermotogae, Actinobacteria, Spirochaetes, Caldiserica, Nitrospirae, Bacteroidetes,* candidate divisions TM6 and OD1, *Synergistetes, Elusimicrobia,* candidate division TM7 and *Acidobacteria* were present as less abundant populations. *Chlorobi,* candidate division OP11, *Deferribacteres, Fibrobacteres*, *Cyanobacteria*, *Armantimonadetes*, candidate division TA06, SR1, etc. were found as minor groups indiscriminately.

*Proteobacteria* was the single most abundant phylum in GR3 (78%) and also the second most abundant group in DB2 (19%), while it contributed only 6.5% in GR1. Members of *Delta*, *Beta* and *Gamma* sub-divisions constituted nearly 99% of *Proteobacteria* in GR3 (Fig. 3b). GR1 and DB2 exhibited a near similar composition with *Firmicutes, Chloroflexi*, OP8, *Thermotogae*, *Actinobacteria, Caldiserica, Nitrospirae, Bacteroidetes* and *Alphaproteobacteria* as the major contributors of respective communities (Fig. 3a). *Anaerolinae, Dehalococcoidetes,* Ellin6529*, Thermomicrobia* etc., of phylum *Chloroflexi* and *Clostridia* of *Firmicutes* constituted major bacterial groups in both GR1 and DB2 (Fig. 3b)*. Thermotogae* (of phylum *Thermotogae*) was a dominant (13%) group in GR1, but also present at moderate abundance in DB2 and GR3 (4.3 and 2.5%, respectively).

**Fig. 4:**
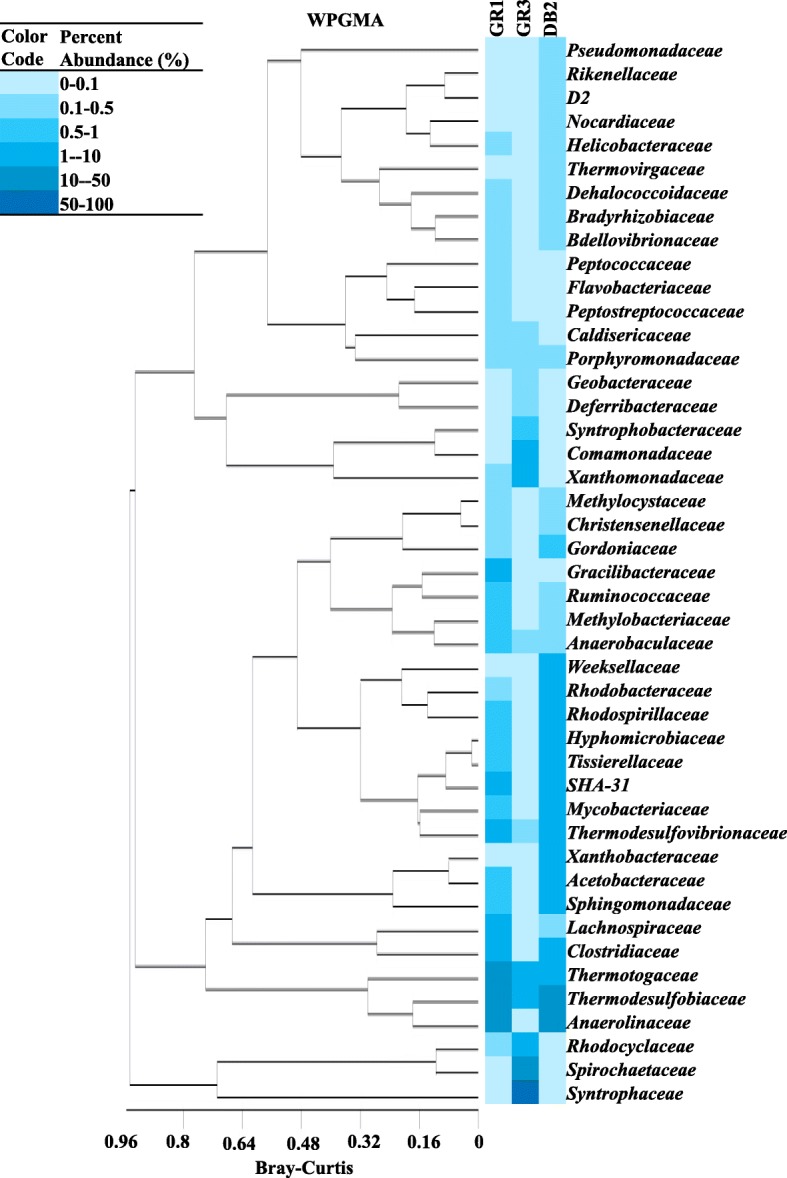
Heat map indicating the relative abundance of major families with cumulative abundance of > 0.2% on the basis of WPGMA (Weighted Pair Group Method with Arithmetic Mean)

Taxonomic distribution at lower levels showed that although a considerable proportion (97%) of GR3 reads could be assigned to family level (compared to 65–69% in GR1 and DB2), genus level assignment of was achieved only for 35% of GR3 reads (49–57% in GR1 and DB2). A total of 418 genera were detected and out of these 140 were common across the three samples. Figure 4 represented the relative abundance of major families across the samples as a semi-quantitative heat map (represented in the text as families (genus); families having ≥0.2% cumulative abundance was considered). WPGMA of these major families present in all three samples showed presence of three broad, yet distinct clades suggesting the correlation among these taxa. *Syntrophaceae* (*Syntrophus*) of *Deltaproteobacteria* was found to be the single most dominant group (> 60% abundance) in GR3, correlated with two other major groups of GR3 namely *Spirochaetaceae* (*Treponema*) (12%) and *Rhodocyclaceae* (*Azovibrio*, *Thauera, Petrobacter* and *Dechloromonas*) (10%). *Thermodesulfobiaceae* (*Coprothermobacter* and *Thermodesulfobium*) of *Firmicutes*, *Anaerolineaceae* (T78 and *Anerolinea*) of *Chloroflexi* and unclassified OP8 members represented the most abundant populations in GR1 and DB2. In contrast to GR3 and DB2, GR1 community harbored considerable proportion (15%) of *Thermotogaceae* (*Fervidobacterium*) a member of *Thermotogae*, *Sphingomonadaceae* (*Kaistobacter*)*, Acetobacteriaceae* (*Acidocella, Acidisoma, Acidiphilum, Acetobacter,* etc) and *Hyphomicrobiaceae* (*Hyphomicrobium, Rhodoplanes, Parvibacculum*) were detected in both DB2 and GR1 with moderate-low abundance (5–0.8%). Noticeably, members of *Xanthomonadaceae* (*Pseudodoxanthomonas, Stenotrophomonas,* etc)*, Commamonadaceae* (*Thiomonas, Acidovorax, Hydrogenophaga,* etc) and *Syntrophobactereaceae* (*Syntrophobacter, Desulfoglaeba, Desulforhabdus,* etc) were present with relatively higher abundance in GR3. There were a number of other families detected with relatively lower abundance in both GR1 and DB2 samples. The family (genus) level community profiles indicated distinctiveness in GR3 leaving DB2 and GR1 more closely related to each other.

**Fig. 5:**
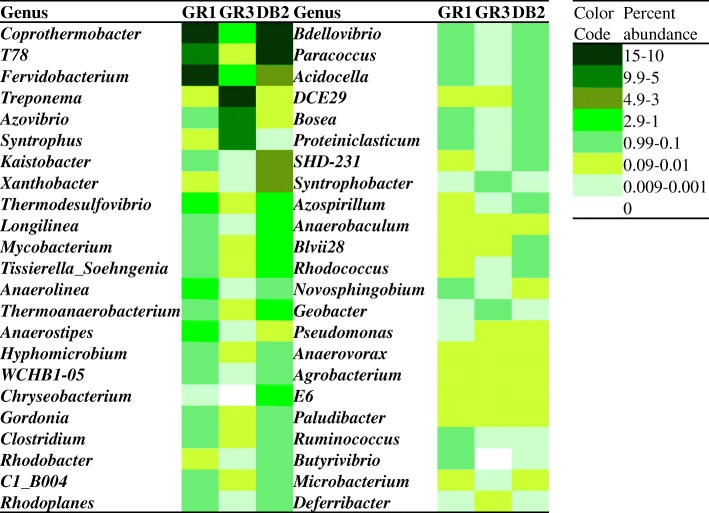
Heat map indicating the relative abundance of major genera with cumulative abundance of > 0.1%

Abundance of genera (cumulative abundance ≥0.1%) within the test sludge samples were depicted in Fig. 5. GR1 sample showed presence of *Coprothermobacter* (14.99%), *Fervidobacterium* (12.68%), T78 (9.53%) as abundant groups. *Anaerostipes* (1.45%), *Thermodesulfovibrio* (1.07%), *Anaerolinea* (1.05%), etc., were also found. Several other taxa (e.g. *Tissierella_Soehngenia*, *Mycobacterium*, *Hyphomicrobium*, *Clostridium*, WCHB1–05, *Gordonia*, *Azovibrio*, *Paracoccus*, *Novosphingobium*, etc.) with minor abundance were present in GR1. In GR3 sample the major taxa found were: *Treponema* (11.87%), *Azovibrio* (9.3%)*, Syntrophus* (6.57%), *Coprothermobacter* (2.88%), and *Fervidobacterium* (2.55%). Presence of *Sytrophobacter* (0.27%), *Geobacter* (0.16%)*,* etc., was noted. The sample from Digboi refinery (DB2) showed a near similar composition to that of GR1 recovered from Guwahati refinery. Members of T78 (14.18%), *Coprothermobacter* (12.51%), *Kaistobacter* (4.71%), *Fervidobacterium* (4.31%), *Xanthobacter* (3.06%), *Longilinea* (2.36%), *Mycobacterium* (1.76%), *Anaerostipes* (1.45%), *Tissierella_Soehngenia* (1.35%), *Clostridium* (1.22%), etc. were detected in DB2. Many other genera like *Acetobacterium, Dietzia, Moorela, Methanobacterium, Methylosinus, Methylobacterium, Methanosaeta,* etc. were present as less abundant members (cumulative abundance < 0.1%) across the samples (Additional file 7: Figure S4).

### Analysis of abundant OTUs

**Fig. 6:**
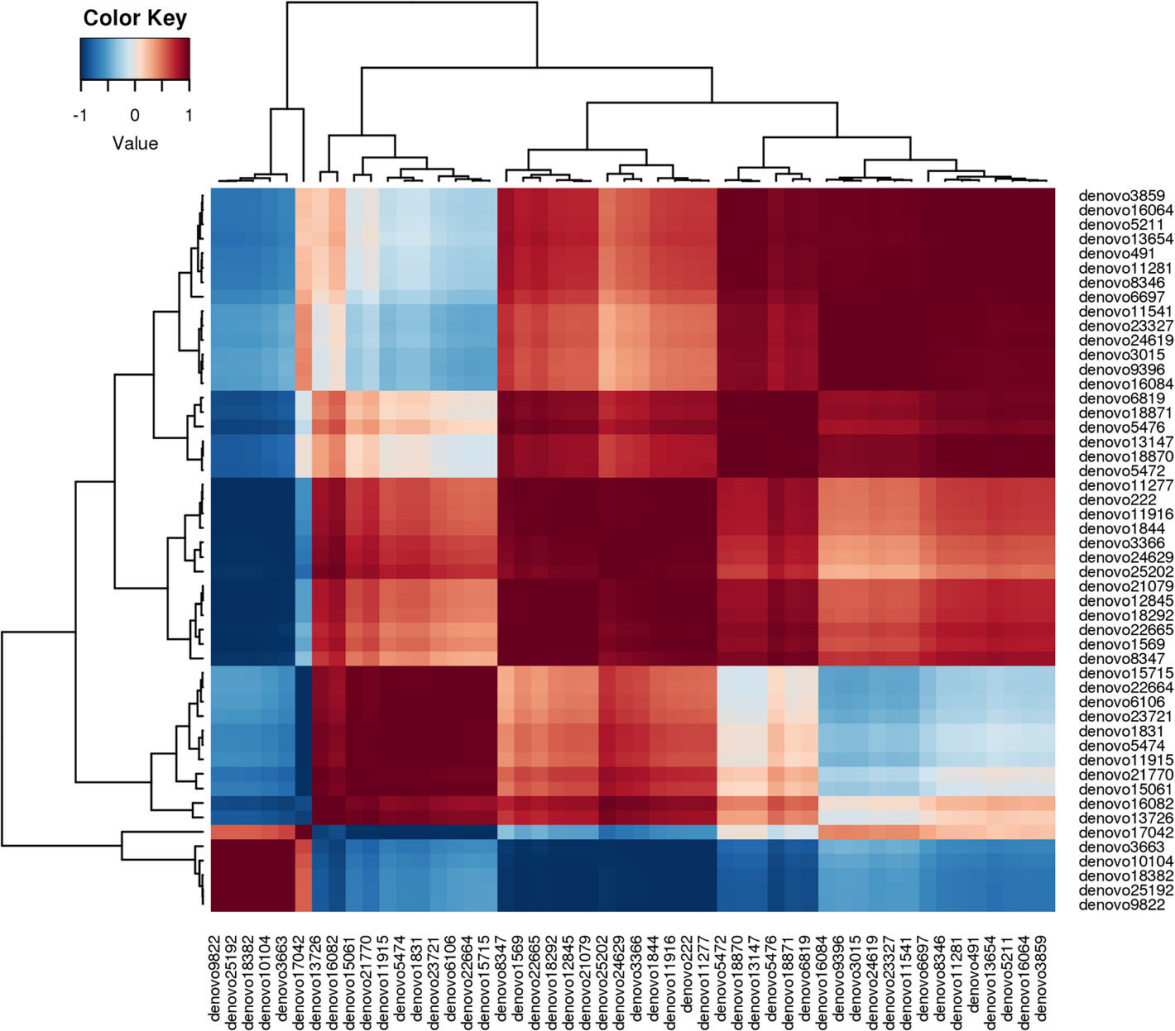
Heat map and UPGMA indicating the Pearson co-efficient of top 50 most abundant OTUs across the samples

Correlation and phylogenetic lineages of de novo OTUs abundant across the three samples were analyzed. Top fifty abundant OTUs (covering 76–84% of total quality reads) were selected and Pearson correlation among their individual abundances was calculated together with UPGMA (Unweighted Pair Group Method with Arithmetic Mean) (Fig. 6). Correlation pattern among these OTUs exhibited four distinct clusters. Phylogenetic lineages of OTUs from each of these four clusters were determined (Additional files 8, 9, 10, 11: Figures S5-S8). The first clade comprised of 21 OTUs representing 53%, 34% and 4% of reads from GR1, DB2 and GR3 respectively showing their lineage with anaerobic, sulfate-reducing organisms having a syntrophic association with methanogens. Taxonomically, these OTUs were mostly affiliated to members of *Firmicutes* (*Coprothermobacter, Clostridiales* members) and *Chloroflexi* (*Anaerolineae*) (Additional file 8: Figure S5). The second clade of 12 OTUs covered 26%, 19% and 2% of total reads from DB2, GR1 and GR3, respectively. OTUs of this clade were phylogenetically related to anaerobic, thermophilic, sulfate-reducing *Thermodesulfovibrio, Anaerolineae*, OP8 and a few members of *Actinobacteria* (Additional file 9: Figure S6).The third clade comprised of 11 OTUs (24%, 2% and 0.04% of DB2, GR1 and GR3 respectively) that showed lineages with mostly sulfate reducing, N_2_ fixing and syntrophic bacteria from hydrocarbon contaminated sites (Additional file 10: Figure S7). The last clade comprised of 6 OTUs (73.37%, 0.98% and 0.76% of GR3, GR1 and DB2, respectively) which were phylogenetically related to organisms capable of N_2_ fixation, sulfate-reduction and/or syntrophic metabolism (Additional file 11: Figure S8).

### Comparison of microbial communities of different petroleum hydrocarbon contaminated environments

**Fig. 7:**
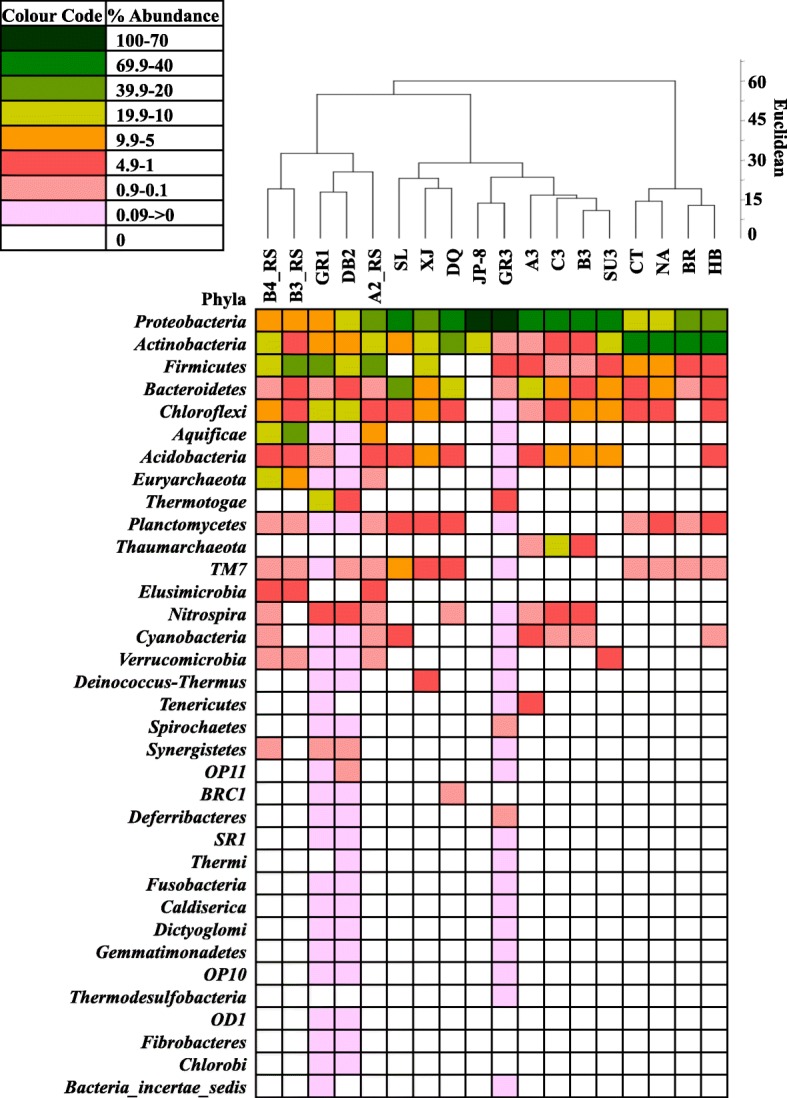
Comparison of microbial communities of different petroleum hydrocarbon contaminated environments on the basis of their phylum distribution

Microbial communities of the test samples were compared with that of fifteen other previously reported petroleum hydrocarbon contaminated environments (Additional file 12: Table S4). Relative abundance of the major phyla present in each sample was plotted as a heat map (Fig. 7). *Proteobacteria* was found as an omnipresent phylum with more than 50% relative abundance in seven out of total eighteen samples. Average abundance of this phylum was 36%. *Actinobacteria, Firmicutes*, *Bacteriodetes* and *Chloroflexi* were also found to be universally occurring taxa with varying abundance. Average abundance of these four taxa was in the range of 4–19%. *Euryarchaeota* was found to be a prevalent archeal taxon in several samples including the test ones. The UPGMA based on Euclidean similarity indices showed three distinct clades among the samples. The first clade was comprised of refuelling station samples (B3_RS and B4_RS) and the test refinery sludge samples DB2 and GR1. Second clade was comprised of oil exploration site samples (XJ, DQ and SL), oil contaminated sediments (A3, B3 and C3), fuel spill Arctic soil (JP-8) and tailing ponds sample (SU3) along with test refinery sludge GR3. Finally, the third clade was comprised of Hubai oil field samples (HB) with naturally attenuated (NA) and bioremediated soil (BT) contaminated with oil. GR1 and DB2 samples were closely associated within the dendogram along with the refuelling station samples because of relatively higher *Firmicutes* (average around 20.4%) and comparatively lower abundance of *Proteobacteria* (average around 12%)*.* GR3 was grouped with oil contaminated soil and sediment due to high abundance of *Proteobacteria* (average around 57%).

### Analysis of archaeal populations

**Fig. 8:**
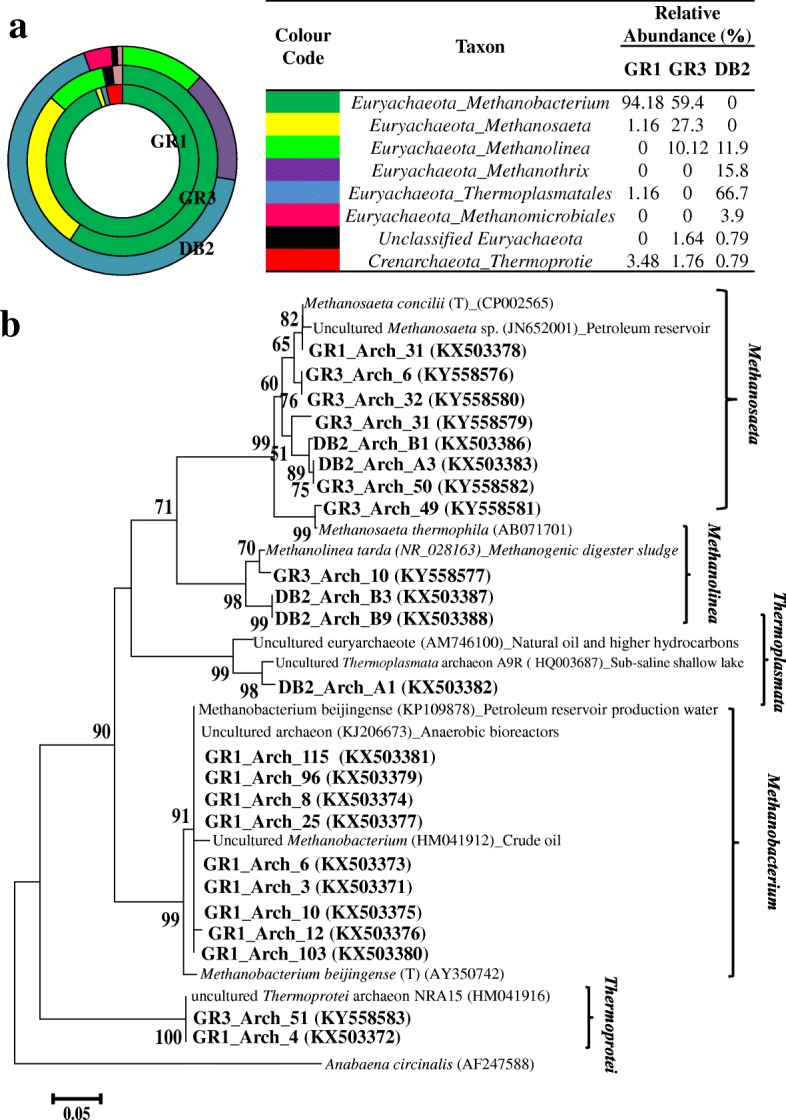
Archaeal distribution and phylogenetic tree of archaeal 16S rRNA gene sequences retrieved from the samples. Distribution of archaebacterial taxa in GR1, GR3 and DB2 (**a**) using clone library analysis. Phylogentic tree was constructed using the neighbour joining method incorporating Jukes-Cantor distance corrections (**b**). Sequence of *Anabaena circinalis* was used as the out-group. One thousand bootstrap analyses were conducted and bootstrap values > 50% were indicated at the nodes. Scale bar = 0.05 change per nucleotide position

The composition of archaeal populations was delineated by analyzing archaeal 16S rRNA gene clone libraries. Total 332 clones from three samples were analyzed. Sequence analysis of all major and several minor (< 1% abundance) OTUs (ARDRA phylotypes) revealed the dominance of the phylum *Euryarchaeota* (Fig. 8a), which was mostly represented by families *Methanobacteriaceae* (genus *Methanobacterium*) in GR1 and GR3, *Methanosaetecae* (*Methanosaeta*) in GR3, *Methanoregulaceae* (*Methanolinea*) in DB2 and GR3 and class *Thermoplasmata* predominantly in DB2. Phylum *Crenarchaeota,* present as the relatively minor population in all three samples, was represented by unclassified *Thermoprotei* members.

Phylogenetic lineages of archaeal sequences were studied (Fig. 8b). Sequences affiliated to *Methanobacterium* were closely related to methanogenic *Methanobacterium beijingense* type strain 8–2; isolated earlier from the anaerobic digester [49]. Members of *Methanosaeta* showed their close relatedness with *M. concilii*, which is a “specialist in acetoclastic methanogenesis” [50]. A single OTU from GR3 also showed affiliation to *M. thermophilia* [51]. A few OTUs from DB2 and GR3 were distantly related to the *Methanolinea* clade but showed their closeness with *Methanolinea tarda*, a novel methane producing strain originally obtained from a mesophilic methanogenic sludge digesting municipal sewage sludge [52]**.** Clones representing the class *Thermoprotei* showed phylogenetic relatedness with uncultured *Thermoprotei* archaeon NRA15 reported earlier from microbial communities associated with crude oil, large insoluble particles and formation water components of the reservoir fluid from a non-flooded high-temperature petroleum reservoir [15].

### PICRUSt analysis

**Fig. 9:**
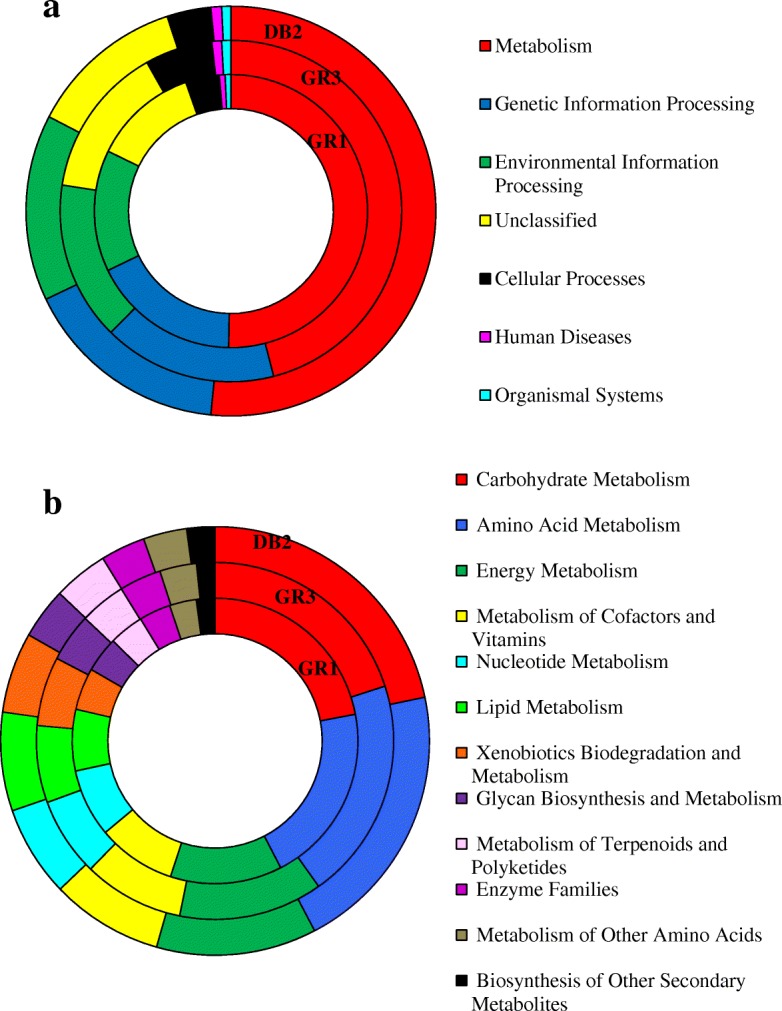
PICRUSt based analysis of functional potential of three communities. Overall distribution of the functional inventory in GR1, GR3 and DB2 (**a**). Distribution of genes involved in metabolism within the samples (**b**)

PICRUSt analysis was done to predict the putative metabolic properties of microbial communities in the samples. For better insight, we have split the taxonomic assignment data into archaeal and bacterial reads separately. The NSTI for the bacterial library in all three samples varied from 0.18 to 0.11 in GR1, DB2 and GR3 respectively. Broad classes of KEGG pathways predicted in each of the samples are presented in Fig. 9a. Genes related to metabolic activities accounted for almost 46–52% of entire genetic repertoire in GR3, GR1 and DB2 metagenome.

The most abundant group of metabolism-related genes in three metagenomes were those involved in carbohydrate- (20% to 22% of all the genes involved in metabolism) followed by amino acid- (20.3% to 20.6%) and energy- metabolism (12.09% to 13.03%) (Fig. 9b). The predicted bacterial metagenomes of GR3 differed mostly from the rest two with respect to different sub-categories of gene system for metabolism. Gene prediction on carbohydrate metabolism revealed a relatively higher degree of allocation of genes related to butanoate, glyoxylate and dicarboxylate metabolism in GR3 compared to the other two samples (Additional file 13: Table S5), while the genes allocated for glycolysis/gluconeogenesis, TCA cycle, propionate and pyruvate metabolism was found to be less in GR3 than GR1 and DB2. With respect to amino acid metabolism, amino acid related enzymes (12.53% to 14.64% of all amino acid metabolism genes), followed by genes encoding arginine and proline metabolism were abundant (Additional file 13: Table S5). The latter, along with the genes for cysteine and methionine metabolism were less abundant in GR3. With respect to energy metabolism, oxidative phosphorylation-related genes were highly abundant in GR1 and DB2 followed by genes for CO_2_ fixation in prokaryotes. However, the latter system was most abundant in GR3. Genes for methane and nitrogen metabolism were present in all three samples with considerable abundance (with slightly higher values in GR3). Interestingly, genes related to photosynthesis were relatively more frequent in GR1 and DB2 rather than GR3. This fact about photosynthetic ability of GR1 and DB2 communities corroborated very well with abundant anoxic photosynthetic green non-sulfur bacteria like T78 and WCHB1–05 in these two samples. Among xenobiotic degradation, genes related to xylene, nitrotoluene, naphthalene and benzoate degradation were observed more in GR3 as compared to GR1 and DB2. Certain xenobiotic degradation genes like, toluene, flurobenzoate, styrene, atrazine, bisphenol, chloroalkene, etc. were observed more abundantly in DB2. Among lipid metabolizing genes, lipid biosynthetic proteins were on the top of the list. Interestingly, genes related to fatty acid biosynthesis were more in GR1 and DB2 than GR3 while fatty acid metabolism was highest in GR3 and lowest in GR1. Among the genetic systems for environmental information process, genes related to membrane transport (including transporter, ABC transporters and secretion system) were most abundant followed by those involved in signal transduction (majorly two-component systems). Genes under the genetic information processing category were mostly related to DNA repair and recombination proteins, ribosomes, aminoacyl tRNA biosynthesis, transcription factors etc., involved in several processes of replication, repair, translation, transcription, followed by sorting and degradation.

### Analysis of functional genes

**Fig. 10:**
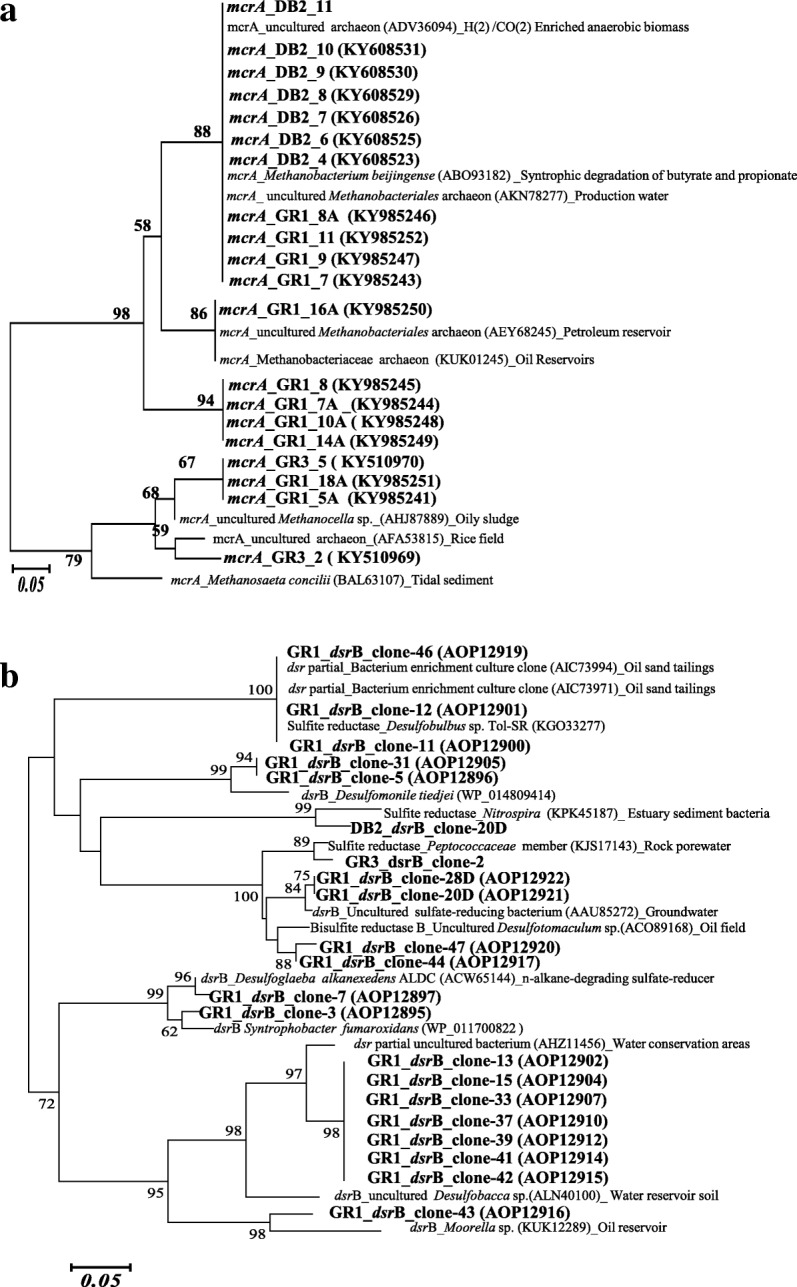
Phylogenetic tree of functional genes *mcr*A (**a**) *dsr*B (**b**) based on translated partial amino acid sequences. Trees were constructed using neighbor joining method and bootstrap values > 50% were indicated at the nodes. One thousand bootstrap analyses were conducted. Scale bar = 0.05 change per amino acid position

Quantitative real-time PCR was used to determine the abundance of *mcr*A and *dsr*B genes. These two genes were targeted considering the potential role of methane metabolizing and sulfate-reducing populations within the studied communities. Nearly equal distribution (~ 10^6^ gene copies g^− 1^ sample) of the *mcr*A gene in all the three samples was noted. In contrast, abundance of the *dsr*B gene was found to be relatively higher in GR1 and DB2 (10^8^ gene copies g^− 1^ sample) compared to GR3 (10^7^ gene copies g^− 1^ sample) (Table 1). Phylogenetic analysis based on derived amino acid sequences of *mcr*A gene indicated a close relatedness to *Methanobacterium beijingense* and uncultured *Methanobacteriales* retrieved from petroleum reservoir or production water, syntrophic organisms capable of degrading butyrate and propionate, obligate hydrogenotrophic methanogen *Methanocella* and acetoclastic methanogen *Methanosaeta* (Fig. 10a). Abundance of *Methanobacterium* in the sludge samples were also reported from clone library of archaeal 16S rRNA gene. Phylogenetic analysis of *dsr*B gene revealed the lineages of *dsr*B genes with two major taxonomic domains of the communities, namely the *Deltaproteobacteria* and *Firmicutes* along with *Nitrospirae* members. Particularly, the sequences showed close affiliations to *dsr*B sequences from *Desulfobacca, Syntrophobacter, Desulfoglaeba, Desulfomonile* and *Desulfobulbus* of *Deltaproteobacteria; Desulfotomaculum, Moorella* and *Peptococcaceae* of *Firmicutes* and *Nitrospirae* (Fig. 10b).

## Discussion

The present study elucidated microbial ecology of three hydrocarbon enriched refinery wastes providing better insights into the community structure, metabolic potential of major populations and the complex interrelations among the populations. Strong reducing condition coupled with lack of disolved oxygen, adequate moisture, nitrogen and phosphorus levels of the samples corroborated well with the characteristic nature of hydrocarbon-rich, anoxic oily sludge and hydrocarbon resource environment [10, 30, 42, 53]. According to the Peters-Moldowan scale on quantitative estimation of the mass of oil degraded which suggests that a loss of up to 50% of C_6_ and above compounds could be correlated to the level of biodegradation [54]. Following this condition, the observed abundance of C_12_-C_20_ compounds in our samples indicated a poor level of biodegradation. Lack of appreciable hydrocarbon bioattenuation (rate and extent of degradation) could be due to one or multiple reasons including paucity of nutrients and other chemicals necessary for microbial metabolism or activation of the hydrocarbons [10, 13, 30]. The test samples were found to be rich in medium or long chain alkanes with branching and substitutions. Most of these hydrocarbons have been reported to be least reactive to oxidation as activation of their C-H bonds requires various abiotic factors including high temperature or pressure, UV light or chemical oxidant. [55]. Alvarej and Illman, [56] reported that branching and substitutions increase recalcitrance. Microbial degradation of these compounds requires either oxygen or nitrate, which serves as a reactant for alkane activation and as a TEA. Lack of dissolved oxygen in the present waste could impair the oxygenic activation process and also reduce the overall metabolic requirement due to shift in electron transport processes. Under anaerobic condition, a narrow range of alkane is metabolized by sulfate or nitrate-reducing bacteria and activation of alkanes is done by fumarate addition [55, 57]. Although relatively higher amounts of sulfate were found in GR1 and DB2, thermodynamically anaerobic hydrocarbon biodegradation coupled with sulfate reduction (dissimilatory sulfate reduction) is considered to be less favored than nitrate or nitrite reduction [58]. In contrast, assimilatory and/or dissimilatory reductions of nitrate or nitrite often facilitate oxidation of organic compounds [6, 59]. In microaerophilic and anaerobic conditions, nitrate is not only used as TEA (Terminal Electron Acceptor) but also act as an activator of the alkanes facilitating their biodegradation [55, 58]. High TPH containing anoxyic, mesophillic to slightly thermophillic nature of the test sludge samples resembled with waste generated from various other refineries and oil storage facilities [1, 4, 6, 10, 42, 60]. The prevailing conditions could favour growth of methanogenic microorganisms which are on the extreme end of thermodynamic benefits and often depend on metabolic products of anaerobic hydrocarbon degradation (acetate, CO_2_ and H_2_) produced by bacterial counterpart [15]. However, lack of nitrate, the thermodynamically favoured electron acceptor over sulfate in such oxygen deficient environment impairs the intrinsic bioremediation potential of native microbiome [54, 58, 61].

Microbiological diversity and other related quantitative parameters (e.g.*,* total cell counts and 16S rRNA gene copy numbers) indicated that in spite of high TPH content and insufficient N and P nutrient, the waste sludge harbored rich microbial communities with considerable species diversity and cell abundance. The values of diversity indices, as well as total and cultivable cell counts were comparable with those of various other petroleum producing/contaminated environments (Additional file 4: Table S3) [8, 22, 62]. Relatively higher values of the Shannon indices for GR1 and DB2 samples in particular corroborated well with that of refuelling station, oil field and soil contaminated from abandoned oil wells samples (5.5–8.5). The Shannon index of GR3 was slightly low, which could be attributed to the extreme nature with respect to higher temperature and hydrocarbon content of the sample and was found to be in line with production water, injection water and beach sand exposed to deep water horizon spill (3.4–5.2). Phylum level distribution indicated that GR1 and DB2 sludge samples showed resemblance with that of refuelling station. The qPCR analysis indicated an abundance of microbial cells (bacteria and archaea). The copy number of *dsr*B gene in GR1 (2.2 × 10^8^/g) or DB2 (3.5 × 10^8^/g) was almost 10 times higher than that of GR3 (4 × 10^7^/g). This observation corroborated well with comparatively higher abundance of sulfate-reducers like *Coprothermobacter, Thermodesulfovibrio,* etc. in GR1 and DB2 than in GR3. Abundance of archaea, in particular could be attributed to the anaerobic, hydrocarbon-rich state of the samples that lack dissolved O_2_ and other inorganic electron acceptors and catabolically rely on a restricted number of simple compounds, e.g., CO_2_ as an oxidant with H_2_ as an electron donor or on acetate, methanol and formate [63]. The observed microbial abundance within these sludge samples presented a contrast to the perception that unlike natural environment with high microbial diversity, hydrocarbon-rich environment containing relatively limited variety of carbon sources will support a lower microbial diversity [21]. Our observation suggests that the inherent toxic effect of hydrocarbons, improper nutritional condition and other physical constraints could not diminish the development of microbial community within refinery sludge. From the intrinsic bioremediation feasibility point of view, a diverse community is preferred, as higher diversity means that a given process could be carried out under a broader range of environmental conditions.

Our study illustrated the community composition of refinery sludge samples and explored the complexity of interactions within the various guilds. All the three samples were found to be mainly colonized by hydrocarbon metabolizing strict anaerobic populations along with a few aerobic bacteria as minor groups. The predominance of *Chloroflexi, Firmicutes, Deltaproteobacteria, Thermotogae* and *Methanobacteria* was in accordance with oxygen limited, hydrocarbon-rich, reduced (negative ORP) state of the samples. Members of *Chloroflexi* have been reported to be involved in the fermentative metabolism of alkanes under anaerobic sulfate-reducing condition and even connected to methanogenesis through reverse electron transport [63, 64]. Role of strictly anaerobic *Deltaproteobacteria* members (e.g.*, Syntrophus, Syntrophobacter, Geobacter,* etc.) in biodegradation was undoubtedly proven. Members of this taxa have been implicated as one of the most potent group involved in activation and subsequent oxidation of broad range alkanes via long chain fatty acids (LCFA) metabolism to acetate and hydrogen in methanogenic environments [24, 65]. Presence of *Firmicutes* in anaerobic hydrocarbon degrading communities as a primary biodegrading population has been previously reported [24, 25]. Strict anaerobic members of this phylum are known for biodegradation of broad ranges of alkanes and iso-alkanes under sulfate reducing or methanogenic conditions [66, 67]. Especially, the abundance of anaerobic, fermentative, thermophilic *Coprothermobacter* (*Firmicutes*) capable of producing acetate/H_2_ and maintaining of syntrophic association with hydrogenotrophic archaea could be noticed. *Coprothermobacter* was previously implicated in different anaerobic biodegradation pathways in diverse mesophilic to thermophilic anaerobic sludge [68, 69]. Predominance of hydrogenotrophic *Methanobacterium*, *Methanocella*, etc. as the major archaeal populations but fewer acetoclastic methanogens (e.g. *Methanosaeta*) are in good agreement with the presence of syntrophic, fermentative (mainly hydrogenotrophic), sulfate-reducing bacterial populations. Methanogenic archaea are known to co-exist with fermentative, sulfate-reducing and syntrophic organisms producing simple substrates like CO_2_, H_2_, acetate, formate, methanol, etc. [70]. Hydrogenotrophic methanogens maintain low hydrogen concentration within methanogenic hydrocarbon-rich environment thus facilitating the growth of fermenting organisms and hydrocarbon degradation. The abundance of methanogenic archaea and methanogenesis process within the sludge environment was supported by the existence of functional biomarkers *mcr*A gene. Together with strict anaerobic populations, minor presence of aerobic–microaerophilic groups in refinery wastes corroborate with recent findings on microbial communities in hydrocarbon resource environments [9, 30]. Presence of aerobic/facultative anaerobic, nitrate reducing *Novosphingobium, Paracoccus* and *Hyphomicrobium* (*Alphaproteobacteria*) was in accordance with previous reports that indicate enhanced denitrification in the presence of methanol and implicate the role of these hydrocarbon metabolizing organisms in the petroleum-rich environments [71–73]. N_2_ fixing *Azovibrio* (*Betaproteobacteria*), *Azospirillum* and *Rhodobacteria* (*Alphaproteobacteria*) found in nitrogen deficient refinery waste could facilitate the supply of fixed nitrogen essential for community function. Members of *Rhodobacteraceae* were capable of methane oxidation (forming methanol) and thereby support the metabolism of methanol-utilizing populations [72].

PICRUSt analysis highlighted diverse and complex assemblages of genes related to hydrocarbon degradation, nitrate/sulfate metabolism, fermentation and methane metabolism. Most of the genes were involved in survival processes of bacteria in this particular environment. Varied distribution of genes involved in degradation of xenobiotic compounds was observed among the samples with slightly higher in GR3 for xylene, nitrotoluene, naphthalene and benzoate degradation and DB2 for styrene, toluene, bisphenol, fluorobenzoate, chloroalkane and chloroalkene degradation. The predominance of transporter genes especially those involved in the ABC-type transport system which are often present in gene clusters connected to aromatic compound metabolism were observed [36]. Overall, PICRUSt analysis highlighted the degradation potential of wide range of aromatic and aliphatic hydrocarbons and supported the functional group distribution which is in accordance with phylogenetic distribution in such petroleum hydrocarbon-rich environment.

Our observations on microbial ecology of refinery waste sludge highlighted the potential of communities towards mineralization of hydrocarbons through a concerted effort of diverse microbial populations forming functional guilds. Under reducing environment, mineralization of organic compound could be more complex and may require cooperation of different groups of residing microorganisms [34]. Previous investigators have reported *Pseudoxanthomaonas, Mycobacterium, Gordonia, Microbacterium,* etc., as aerobic hydrocarbon degraders, *Novosphigobium*, *Pseudomonas, Bacillus, Dietzia,* etc. as facultative anaerobic degrader and *Longilinea, Geobacter*, etc. as obligate anaerobic degraders which were also observed in our samples as major microbial groups [6, 9, 30, 42, 61, 71, 73–75]. It was also been reported that such microorganisms could degrade long chain aliphatics and aromatics into smaller hydrocarbons, which could be further degraded into small acids (butyrate, propionate, acetate, formate, etc.), alcohols (ethanol, methanol, etc.), CO_2_ and H_2_ by fermentative microorganisms including *Coprothermobacter*, *Anaerostipes, Paludibacter, Anaerobaculum*, *Clostridium, Anaerovorax*, *Syntrophus,* etc., as also observed in test sludge samples [25, 69, 76–81]. Rojo et al., [57] reported that in oxygen deficient environment, alkanes could be metabolized by sulfate- or nitrate-reducing bacteria by activating alkanes through the addition of fumarate. Within sludge the community many microbial groups known to be sulfate/thiosulfate- (*Coprothermobacter*, *Anaerobaculum*, *Thermodesulfovibrio* etc.), nitrate- (*Gordonia*, *Novosphigobium*, *Bacillus,* etc.), iron- (*Geobacter*) and managanese- (*Dietzia*) reducer were present [6, 42, 61, 70, 71, 77–79]. It was known that acetate produced by the fermentative organisms could undergo oxidation by syntrophic acetate oxidizers like *Clostridium* resulting in the formation of CO_2_ and H_2_ [80]. The conversion of resultant CO_2_ and H_2_ into methane by hydrogenotrophic methanogens like *Methanobacterium*, *Methanocella,* etc., was well known phenomenon. Such methanogenic archaea were also found in all the three sludge microbiomes [9, 42, 49, 81]. Acetate could be directly converted to methane by acetoclastic methanogens like *Methansaeta* [50]. Mbadinga et al., [82] stated that formate and hydrogen could also be metabolized by hydrogenotrophic methanogens to methane. In anaerobic environments, methanogenesis could play an important role for the degradation of hydrocarbons in polluted soils, aquifers and oil reservoirs, thus contributing considerably to the mineralization of petroleum hydrocarbons [63]. Methanol produced as fermentation product has been known to be used by methylotrophs like *Methylobacterium, Hyphomicrobium,* etc. [72]. Reports on few species of *Methylobacterium* utilizing methane have been also found [83]. *Methylosinus* a methane oxidizer was also found as a member of sludge microbiome [72]. Varied abundance of these methanogenic, methlytrophic and methanotrophic organisms were observed in all the test sludge samples. The syntrophic association between various fermentative, sulfate-reducing members like *Syntrophus*, *Coprothermobater, Syntrophobacter, Syntrophomonas,* etc. with methanogens have been previously reported from oil associated environment [9, 34, 68, 69, 76]. The close proximity of aerobic and anaerobic bacteria in the petroleum resource environment community was also evident earlier [9]. Although the sludge storage tanks, sampled for this study were grossly anaerobic, our findings suggested presence of both aerobic and anaerobic organisms. The top layer of waste storage tanks were always exposed to air and aerobic bacteria could thrive in such environment. Moreover, convective and diffusive fluxes and rate of reaction of O_2_ with various organic or inorganic targets often determine the concentration of O_2_ within the sludge itself. The samples were rich in aliphatic and aromatic hydrocarbons. The readily degradable fractions (oxidizable) of the constituent hydrocarbons consumes available oxygen as electron acceptor resulting in the production of CO_2_ and other metabolic intermediates, which could further reduce the dissolved oxygen. This might support a cascade of anaerobic metabolism by community members.

## Conclusion

We have explored the microbial community composition of petroleum rich refinery wastes and their catabolic potentials. Refinery sludge microbiomes were comprised of hydrocarbon degrading (*Longilina*, *Mycobacterium, Gordonia, Novosphingobium*, *Geobacter,* etc.) fermentative (*Coprothermobacter*, *Fervidobacterium, Anaerostipes, Anaerobaculum*, *Clostridium, Anaerovorax*, etc.), sulfate-reducing (*Coprothermobacter*, *Anaerobaculum*, *Thermodesulfovibrio,* etc.), syntrophic (*Syntrophus*, *Coprothermobater, Syntrophobacter*, etc.), nitrogen fixing (*Azovibrio, Rhodobacter,* etc.) and methanogenic (*Methanobacterium*, *Methanosaeta, Methanocella,* etc.) microorganisms, which are in accordance with the prevailing physicochemical nature of the samples. Most of the bacteria capable of anaerobic utilization of hydrocarbons could exist in syntrophic alliance with methanogenic organisms that consume their metabolic end products. Methyl coenzyme M reductase A (*mcr*A) and dissimilatory sulfite reductase beta-subunit (*dsr*B) gene phylogeny confirmed methanogenic and sulfate-reducing activities within sludge environment endowed by hydrogenotrophic methanogens and sulfate-reducing *Deltaproteobacteria* and *Firmicutes* members. Overall observation indicated the possibilities of on-site bioremediation of oil refinery sludge exploiting the metabolic interplay of the indigenous microbial populations.

## Additional files

Additional file 1: **Table S1.** Details of PCR primers and PCR conditions. (DOC 39 kb)

Additional file 2: **Table S2.** Detailed hydrocarbon distribution of oily sludge samples. (DOC 73 kb)

Additional file 3: **Figure S1.** Rarefaction curves of the three oily sludge samples (GR1, DB2 and GR3) on the basis of OTUs from V3 region based 16S rRNA amplicon library. (PPTX 42 kb)

Additional file 4: **Table S3.** Comparison of major alpha diversity parameters among various hydrocarbon rich samples. (DOC 76 kb)

Additional file 5: **Figure S2.** Venn diagram of unique and shared taxa. Unique and shared taxa distribution between 3 samples at OTU level and subsequently at phyla, class, family and genus level showed as Venn diagram. (PPTX 270 kb)

Additional file 6: **Figure S3.** Distribution of minor phyla with cumulative abundance of < 0.5. (PPTX 43 kb)

Additional file 7: **Figure S4.** Heat map indicating the relative abundance of minor genera with cumulative abundance of 0.1–0.01%. (PPTX 364 kb)

Additional file 8: **Figure S5.** Phylogentic tree representing of clade1 of top 50 most abundant OTUs. Tree was constructed using the neighbour joining method incorporating Jukes-Cantor distance corrections. One thousand bootstrap analyses were conducted and bootstrap values > 50% were indicated at the nodes. Scale bar = 0.05 change per nucleotide position. The values in bracket indicated abundance in following the sequence of GR1/DB2/GR3. (PPTX 91 kb)

Additional file 9: **Figure S6.** Phylogentic tree representing of clade 2 of top 50 most abundant OTUs. Tree was constructed using the neighbour joining method incorporating Jukes-Cantor distance corrections. One thousand bootstrap analyses were conducted and bootstrap values > 50% were indicated at the nodes. Scale bar = 0.02 change per nucleotide position. The values in bracket indicated abundance in following the sequence of GR1/DB2/GR3. (PPTX 74 kb)

Additional file 10: **Figure S7.** Phylogentic tree representing of clade 3 of top 50 most abundant OTUs. Tree was constructed using the neighbour joining method incorporating Jukes-Cantor distance corrections. One thousand bootstrap analyses were conducted and bootstrap values > 50% were indicated at the nodes. Scale bar = 0.02 change per nucleotide position. The values in bracket indicated abundance in following the sequence of GR1/DB2/GR3. (PPTX 74 kb)

Additional file 11: **Figure S8.** Phylogentic tree representing of clade 4 of top 50 most abundant OTUs. Tree was constructed using the neighbour joining method incorporating Jukes-Cantor distance corrections. One thousand bootstrap analyses were conducted and bootstrap values > 50% were indicated at the nodes. Scale bar = 0.05 change per nucleotide position. The values in bracket indicated abundance in following the sequence of GR1/DB2/GR3. (PPTX 54 kb)

Additional file 12: **Table S4.** Details of different hydrocarbon contaminated samples considered for comparative analysis. (DOCX 12 kb)

Additional file 13: **Table S5.** Detailed distribution of predictive genes within sludge metagenomes using PICRUSt. (DOC 87 kb)

## Abbreviations

AWCD: Average Well Color Development
CLPP: Community level physiological profiling
DO: Dissolved Oxygen
*dsr*B: Dissimilatory sulfite reductase beta-subunit
KEGG: Kyoto Encyclopedia of Genes and Genomes
*mcr*A: Methyl coenzyme M reductase A
NSTI: Nearest Sequenced Taxon Index
ORP: Oxidation Reduction Potential
OTU: Operation Taxonomic Unit
PICRUSt: Phylogenetic Investigation of Communities by Reconstruction of Unobserved States
QIIME: Quantitative Insights into Microbial Ecology
qPCR: Quantitative Polymearse Chain Reaction
TPH: Total Petroleum Hydrocarbon
UPGMA: Unweighted Pair Group Method with Arithmetic Mean
