# Supplementary material for: Petroleum hydrocarbon rich oil refinery sludge of North-East India harbours anaerobic, fermentative, sulfate-reducing, syntrophic and methanogenic microbial populations
Source: BMC Microbiol. 2018 Oct 22;18:151. doi: 10.1186/s12866-018-1275-8 (PMC6198496; doi:10.1186/s12866-018-1275-8)
Supplement: Supplementary file 1 — Table S1. Details of PCR primers and PCR conditions. (DOC 39 kb) [file 12866_2018_1275_MOESM1_ESM.doc]

**Table S1 Details of primers and PCR conditions**

| **Gene** | **Primers (5’-3’)** | **Size (bp)** | **Annealing temperature (°C)** | **Ref** |
| --- | --- | --- | --- | --- |
| **Real Time** |  | | | |
| Bacteria | 341F (CCTACGGGAGGCAGCAG)  518R (ATTACCGCG GCTGCTGG) | 170 | 55 | Muyzer et al., 1998 |
| Archaebacteria | 344F (ACGGGGCGCAGCAGGCGCGA)  744R (CCSGGGTATCTAATCC) | 430 | 55 | Purkamo et al., 2016 |
| *mcr*A | ME1 (GCMATGCARATHGGWATGTC)  ME3 (TGTGTGAAWCCKACDCCACC) | 330 | 55 | Purkamo et al., 2016 |
| *dsr*B | p2060F (CAACATCGTYCAYACCCAGGG)  4R (GTGTAGCAGTTACCGCA) | 450 | 55 | Purkamo et al., 2016 |
| **Clone Library** |  | | | |
| Archaebacteria* | 21F (TTCCGGTTGATCCYGCCGGA)  958R (TTCCGGTTGATCCYGCCGGA) | 940 | 55 | DeLong, 1992 |
| *mcr*A* | ME1 (GCMATGCARATHGGWATGTC)  ME2 (TCATKGCRTAGTTDGGRTAGT) | 790 | 50-55 | Hales et al., 1996 |
| *dsr*B* | p2060F (CAACATCGTYCAYACCCAGGG)  4R (GTGTAGCAGTTACCGCA) | 450 | 56 | Geets et al., 2006, Wagner et al. 1998 |

*All PCR reactions were carried out with denaturation at 94 °C for 5mins, followed by 35 cycles of initial denaturation at 94 °C for 45 s, 30 s at respective annealing temperature and extension at 72 °C for 1 min (in case of gene size >700 bp), 45s (for gene size <700 bp) and a final extension at 72 °C for 10 minutes.
