# Supplementary material for: Petroleum hydrocarbon rich oil refinery sludge of North-East India harbours anaerobic, fermentative, sulfate-reducing, syntrophic and methanogenic microbial populations
Source: BMC Microbiol. 2018 Oct 22;18:151. doi: 10.1186/s12866-018-1275-8 (PMC6198496; doi:10.1186/s12866-018-1275-8)
Supplement: Supplementary file 2 — Table S2. Detailed hydrocarbon distribution of oily sludge samples. (DOC 73 kb) [file 12866_2018_1275_MOESM2_ESM.doc]

**Table S2 Detailed hydrocarbon distribution of oily sludge samples**

| **Hydrocarbons** | **GR1** | **GR3** | **DB2** |
| --- | --- | --- | --- |
| (2s,3r)-3-Dimethyl-T-Butylsiloxy-2,4-Dimethylpentan-1-Ol | 2.97 | 0 | 0 |
| Heptane^$^ | 1.54 | 2.46 | 2.16 |
| Octane^$^ | 1.47 | 0 | 2.04 |
| Nonane^$^ | 1.48 | 0 | 2.01 |
| Decane^$^ | 4.46 | 4.72 | 8.23 |
| 10-Methylhexadecyl Iodide | 1.53 | 0 | 0 |
| Undecane | 1.52 | 0 | 2.11 |
| Dodecane^$^ | 6.03 | 2.39 | 4.2 |
| Tridecane | 0 | 2.4 | 0 |
| Tetradecane | 1.52 | 0 | 2.13 |
| Sulfurous Acid Di-cyclomethyl Ester | 1.42 | 0 | 0 |
| Pentadecane^$^ | 2.99 | 2.38 | 2.13 |
| Hexadecane^$^ | 6.06 | 7.1 | 12.58 |
| Octadecane | 0 | 2.41 | 0 |
| Eicosane | 0 | 2.41 | 2.08 |
| Tricosane- | 6.1 | 14.41 | 23.37 |
| Tetracosane | 4.5 | 4.74 | 0 |
| Pentacosane | 3.04 | 2.38 | 0 |
| Nonacosane | 2.99 | 2.34 | 2.04 |
| Tetratriacontane | 0 | 2.4 | 0 |
| Pentatriacontane | 0 | 7.21 | 2.11 |
| Hexatriacontane | 1.5 | 0 | 0 |
| Tetracontane | 0 | 2.36 | 0 |
| **Non-cyclic Aliphatic Compounds** | **51.12** | **62.11** | **67.19** |
| (1e)-2-(3-Hydroxy-1-Propenyl)-1-(2-Methylpropyl)Cyclopentan-1-Ol | 1.55 | 0 | 2.14 |
| 1,3-Dimethyl-Cyclohexane | 0 | 2.21 | 0 |
| Propyl-Cyclohexane | 0 | 0 | 2.04 |
| 1-Ethyl-2-Methyl-Cyclohexane | 0 | 2.31 | 0 |
| 1-Ethyl-4-Methyl-Cyclohexane | 2.86 | 2.32 | 0 |
| Butyl-Cyclohexane | 1.39 | 0 | 1.93 |
| Cyclohexanepropanol- | 1.36 | 0 | 0 |
| Ethyl-Cyclohexane | 1.44 | 2.34 | 1.98 |
| **Cyclic Aliphatic Compounds** | **8.6** | **9.18** | **8.09** |
| 1,2-Diethyl-Benzene | 1.43 | 0 | 0 |
| 1,4-Dimethyl-Benzene | 2.98 | 2.4 | 4.15 |
| 1,2,4-Trimethylbenzene | 0 | 2.37 | 2.05 |
| (1-Methylethyl)-Benzene | 2.89 | 0 | 2.04 |
| 1-Ethyl-4-Methyl-Benzene | 1.49 | 0 | 2.06 |
| 1-Methyl-2-Propyl-Benzene | 0 | 0 | 2.06 |
| (3,3-Dimethylbutyl)-Benzene | 1.3 | 0 | 1.8 |
| 2,4,6-Tri-Isopropyl-Phenol | 0 | 2.37 | 0 |
| Methylnaphthalene | 1.54 | 4.92 | 2.13 |
| 2-Methyl-Naphthalene | 1.44 | 0 | 0 |
| 1,3-Dimethyl-Naphthalene | 1.48 | 2.41 | 0 |
| 1,4-Dimethyl-Naphthalene | 0 | 2.42 | 0 |
| 1,6-Dimethyl-Naphthalene | 3.07 | 0 | 2.1 |
| 1,7-Dimethyl-Naphthalene | 1.54 | 0 | 0 |
| 1,8-Dimethyl-Naphthalene | 0 | 0 | 2.12 |
| 1-Ethyl-Naphthalene | 1.53 | 0 | 0 |
| 2,3,6-Trimethyl-Naphthalene | 3.02 | 0 | 0 |
| 1,6,7-Trimethyl-Naphthalene | 2.95 | 0 | 0 |
| Decahydro-Naphthalene | 1.51 | 2.4 | 2.1 |
| 1,4-Dimethyl-7-(1-Methylethyl)-Azulene | 1.52 | 0 | 0 |
| 1-Methylene-1h-Indene | 0 | 2.45 | 0 |
| 2-Methyl-3-Ethylbicyclo[3.3.0]Octa-3,6-Diene | 0 | 2.18 | 0 |
| 2-Methylchrysene | 2.94 | 0 | 0 |
| Triphenylene | 3.09 | 0 | 0 |
| 7-Phenylindene | 3.05 | 0 | 0 |
| Phenanthrene | 1.54 | 2.44 | 2.14 |
| Endo-3,4-Dimethylcamphor | 0 | 2.35 | 0 |
| **Total Aromatics** | **40.31** | **28.71** | **24.75** |

^$^ Compounds with its substitutions
