# Supplementary figures and images for: Petroleum hydrocarbon rich oil refinery sludge of North-East India harbours anaerobic, fermentative, sulfate-reducing, syntrophic and methanogenic microbial populations
Source: BMC Microbiol. 2018 Oct 22;18:151. doi: 10.1186/s12866-018-1275-8 (PMC6198496; doi:10.1186/s12866-018-1275-8)

## Slide 1
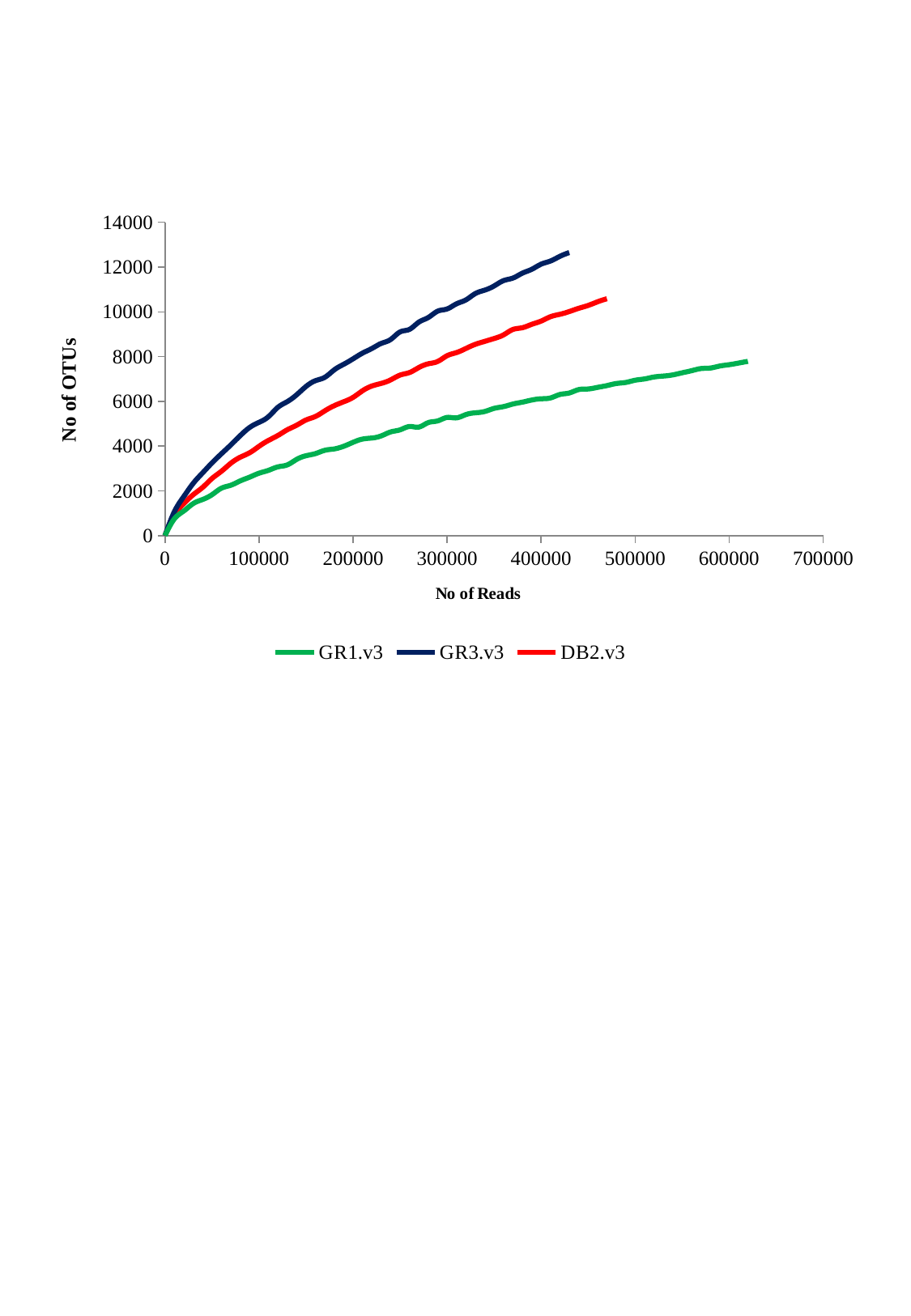

### Chart
| Category | GR1.v3 | GR3.v3 | DB2.v3 |
|---|---|---|---|

Supplement: Supplementary file 3 — Figure S1. Rarefaction curves of the three oily sludge samples (GR1, DB2 and GR3) on the basis of OTUs from V3 region based 16S rRNA amplicon library. (PPTX 42 kb) [file 12866_2018_1275_MOESM3_ESM.pptx]

## Slide 1
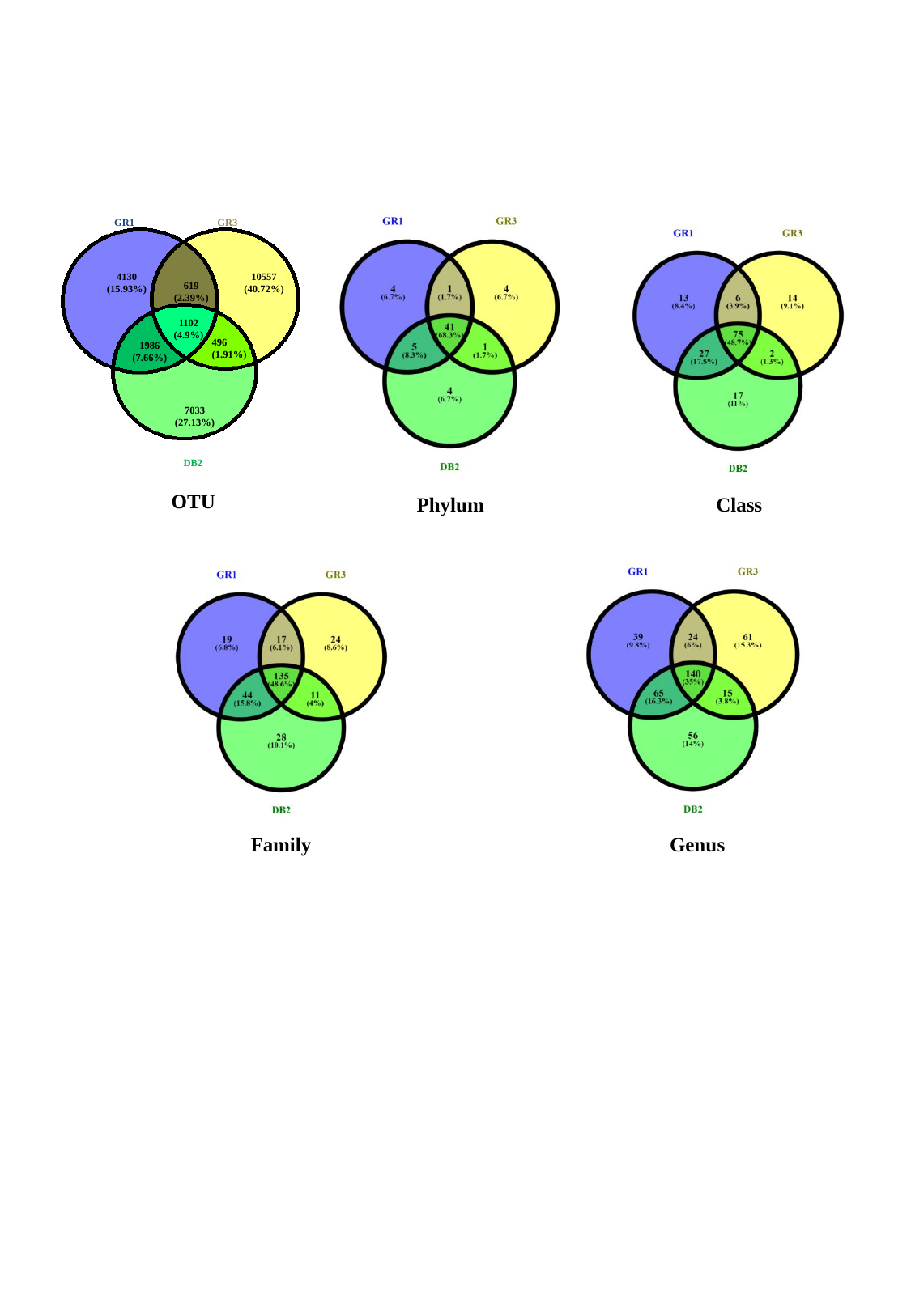

GR1
GR3
4130
(15.93%)
10557
(40.72%)
619
(2.39%)
1102
(4.9%)
496
(1.91%)
1986
(7.66%)
7033
(27.13%)
DB2
OTU
Class
Phylum
Family
Genus

Supplement: Supplementary file 5 — Figure S2. Venn diagram of unique and shared taxa. Unique and shared taxa distribution between 3 samples at OTU level and subsequently at phyla, class, family and genus level showed as Venn diagram. (PPTX 270 kb) [file 12866_2018_1275_MOESM5_ESM.pptx]
