# Supplementary material for: Petroleum hydrocarbon rich oil refinery sludge of North-East India harbours anaerobic, fermentative, sulfate-reducing, syntrophic and methanogenic microbial populations
Source: BMC Microbiol. 2018 Oct 22;18:151. doi: 10.1186/s12866-018-1275-8 (PMC6198496; doi:10.1186/s12866-018-1275-8)
Supplement: Supplementary file 4 — Table S3. Comparison of major alpha diversity parameters among various hydrocarbon rich samples. (DOC 76 kb) [file 12866_2018_1275_MOESM4_ESM.doc]

**Table S3 Comparison of major alpha diversity parameters among various hydrocarbon rich samples**

| **Sample** | **Sequencing Platform** | **Sequencing Details** | **No. of OTUs** | **Shannon** | **Simpson** | **Chao1** | **Coverage (%)** | **Reference** |
| --- | --- | --- | --- | --- | --- | --- | --- | --- |
| GR1 | Illumina | 627638 | 7837 | 6.148 | 0.945 | 15448 | 99.3 | Present Study |
| GR3 | Illumina | 435940 | 12774 | 4.443 | 0.708 | 32314 | 98.2 | Present Study |
| DB2 | Illumina | 472180 | 10617 | 6.396 | 0.95 | 25135 | 98.6 | Present Study |
| Residual oil | Pyrosequencing | 7621 |  | 3.15 | 0.12 | 817 |  | Fowler et al 2016 |
| S (Oil Contaminated soil) | Pyrosequencing | 4365 | 138 | 3.42 |  | 401 |  | Abed et al 2015 |
| OBM (Oil Contaminated soil) | Pyrosequencing | 2347 | 87 | 2.95 |  | 205 |  | Abed et al 2015 |
| UT (Oil Contaminated soil) | Pyrosequencing | 5238 | 166 | 2.61 |  | 611 |  | Abed et al 2015 |
| CS (Oil Contaminated soil) | Pyrosequencing | 957 | 42 | 2.54 |  | 142 |  | Abed et al 2015 |
| B3 (Oil Contaminated soil) | Pyrosequencing | 3750 | 107 | 2.76 |  | 326 |  | Abed et al 2015 |
| JBT60 (The oil contaminated soil from abandoned well mined in 1960s) | Pyrosequencing | 4097 | 1917 | 6.98 | 0.002 | 4832 | 68 | Peng et al 2015 |
| JBT70(The oil contaminated soil from abandoned well mined in 1970s) | Pyrosequencing | 5777 | 1320 | 5.92 | 0.012 | 2602 | 88 | Peng et al 2015 |
| SYT (The oil contaminated soil from recent (one year) oil spilled site) | Pyrosequencing | 2132 | 1161 | 6.7 | 0.0017 | 2838 | 63 | Peng et al 2015 |
| DQ (Oil field) | Pyrosequencing | 26,356 | 1584 | 5.515 |  | 1796 | 94.6 | Liao et al 2015 |
| HB (Oil field) | Pyrosequencing | 20,340 | 1695 | 5.847 |  | 2084 | 90.6 | Liao et al 2015 |
| SL (Oil field) | Pyrosequencing | 32,864 | 1621 | 5.826 |  | 1787 | 98 | Liao et al 2015 |
| XJ (Oil field) | Pyrosequencing | 32,239 | 2100 | 6.185 |  | 2319 | 99.2 | Liao et al 2015 |
| Lu 3064 (Injection well) | Illumina | 16568 | 1238 | 7.07 |  |  | 99.9 | Gao et al 2015 |
| Lu 3084 (Injection well) | Illumina | 19692 | 1197 | 7.11 |  |  | 99.9 | Gao et al 2015 |
| Lu 3065 (Production well | Illumina | 115661 | 1515 | 6.73 |  |  | 99.9 | Gao et al 2015 |
| Lu 3096 (Production well | Illumina | 48400 | 1085 | 6.7 |  |  | 99.8 | Gao et al 2015 |
| A2 (Refuellin Station) | Pyrosequencing | 5,259 |  | 8.59 |  | 3,259 |  | Sutton et al 2013 |
| B3(Refuellin Station) | Pyrosequencing | 8,054 |  | 8.77 |  | 8,348 |  | Sutton et al 2013 |
| B4(Refuellin Station) | Pyrosequencing | 7,636 |  | 8.81 |  | 6,431 |  | Sutton et al 2013 |
| PAH contaminated soil | Clone Library |  |  | 4.87-5.01 |  | 118-938 |  | Martin et al 2012 |
| PNF1 (Prodction Water) | Pyrosequencing | 1398 | 331 | 5.16 |  | 501 |  | Lenchi et al 2013 |
| PNF2 (Prodction Water) | Pyrosequencing | 712 | 121 | 3.35 |  | 243 |  | Lenchi et al 2013 |
| PFS1 (Prodction Water) | Pyrosequencing | 1575 | 178 | 3.66 |  | 360 |  | Lenchi et al 2013 |
| PFOH2 (Prodction Water) | Pyrosequencing | 239 | 11 | 0.94 |  | 14 |  | Lenchi et al 2013 |
| PFOH1 (Prodction Water) | Pyrosequencing | 962 | 206 | 4.04 |  | 526 |  | Lenchi et al 2013 |
| IS2 (Injection water) | Pyrosequencing | 1162 | 199 | 3.82 |  | 472 |  | Lenchi et al 2013 |
| IT3 (Injection water) | Pyrosequencing | 452 | 144 | 4.28 |  | 484 |  | Lenchi et al 2013 |
| IBD (Injection water) | Pyrosequencing | 1567 | 309 | 4.17 |  | 757 |  | Lenchi et al 2013 |
| S1-S9 Soil contaminated with oil | Clone Library |  |  | 1.99-2.8 | 0.082-0.169 |  |  | Thavamani et al 2012 |
| GMR75 | Clone Library |  |  | 3.9925 |  | 153.21 |  | Silva et al 2013 |
| PTS1 | Clone Library |  |  | 3.5952 |  | 104.38 |  | Silva et al 2013 |
| OS-71b (Beach sands exposed to Deep Water Horizon spill) | Pyrosequencing | 7091 | 942 | 4.7 |  |  | 84 | Kostka et al 2011 |
