# Supplementary material for: Petroleum hydrocarbon rich oil refinery sludge of North-East India harbours anaerobic, fermentative, sulfate-reducing, syntrophic and methanogenic microbial populations
Source: BMC Microbiol. 2018 Oct 22;18:151. doi: 10.1186/s12866-018-1275-8 (PMC6198496; doi:10.1186/s12866-018-1275-8)
Supplement: Supplementary file 6 — Figure S3. Distribution of minor phyla with cumulative abundance of < 0.5. (PPTX 43 kb) [file 12866_2018_1275_MOESM6_ESM.pptx]

## Slide 1
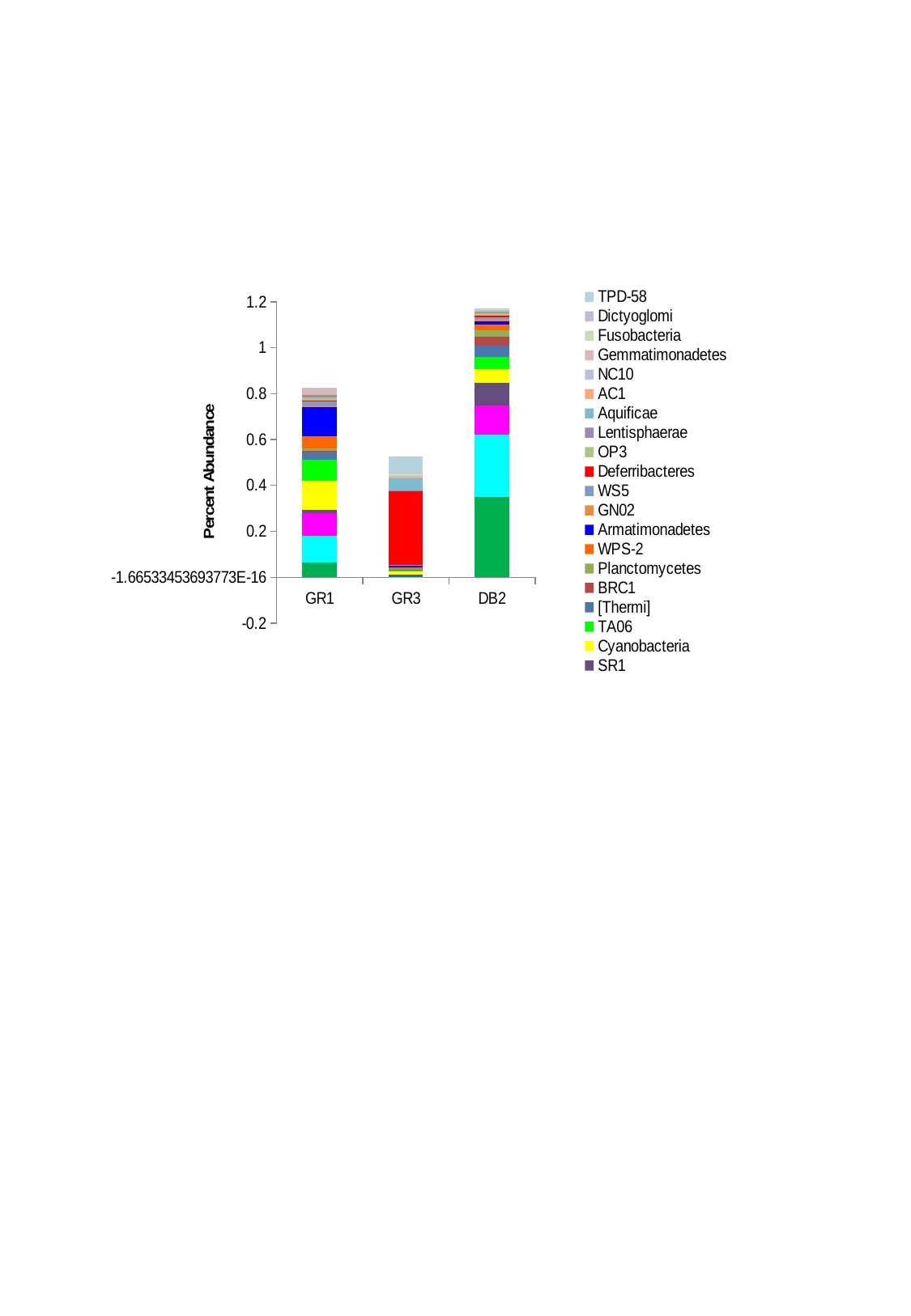

### Chart
| Category | Chlorobi | OP11 | Fibrobacteres | SR1 | Cyanobacteria | TA06 | [Thermi] | BRC1 | Planctomycetes | WPS-2 | Armatimonadetes | GN02 | WS5 | Deferribacteres | OP3 | Lentisphaerae | Aquificae | AC1 | NC10 | Gemmatimonadetes | Fusobacteria | Dictyoglomi | TPD-58 |
|---|---|---|---|---|---|---|---|---|---|---|---|---|---|---|---|---|---|---|---|---|---|---|---|
| GR1 | 0.0654440428391362 | 0.11385927861298696 | 0.10050335150295917 | 0.015025417998781272 | 0.12738215481189014 | 0.09165504979256575 | 0.028882192375435108 | 0.008681352621518068 | 0.011853385310149669 | 0.051253370284731675 | 0.12537876574538595 | 0.0098499962436455 | 0.016194061620908706 | 0.0025042363331302117 | 0.014023723465529186 | 0.01318897802115245 | 0.0008347454443767372 | 0.0026711854220055594 | 0.0008347454443767372 | 0.018698297954038916 | 0.0025042363331302117 | 0.0026711854220055594 | 0.0011686436221274323 |
| GR3 | 0.0020824752763907465 | 0.0004627722836423881 | 0.0 | 0.009024059531026567 | 0.014808713076556419 | 0.004164950552781493 | 0.0046277228364238815 | 0.0009255445672847762 | 0.0009255445672847762 | 0.006941584254635822 | 0.007635742680099403 | 0.0013883168509271644 | 0.0006941584254635822 | 0.32394059854967167 | 0.0 | 0.0004627722836423881 | 0.05252465419341105 | 0.007635742680099403 | 0.0004627722836423881 | 0.003008019843675523 | 0.010643762523774927 | 0.004396336694602687 | 0.0701100009718218 |
| DB2 | 0.35011255955185594 | 0.2715826396523742 | 0.12630228783833305 | 0.10012564787183918 | 0.059988133256548525 | 0.05257141859937525 | 0.04777236793885137 | 0.0394830986161283 | 0.028576165296755843 | 0.022686421304294716 | 0.014833429314346544 | 0.011343210652147358 | 0.008071130656335619 | 0.00785299198994817 | 0.007416714657173272 | 0.005671605326073679 | 0.004362773327748984 | 0.0028358026630368395 | 0.0028358026630368395 | 0.002399525330261941 | 0.0017451093310995934 | 0.001308831998324695 | 0.0006544159991623475 |
