# Supplementary material for: Petroleum hydrocarbon rich oil refinery sludge of North-East India harbours anaerobic, fermentative, sulfate-reducing, syntrophic and methanogenic microbial populations
Source: BMC Microbiol. 2018 Oct 22;18:151. doi: 10.1186/s12866-018-1275-8 (PMC6198496; doi:10.1186/s12866-018-1275-8)
Supplement: Supplementary file 7 — Figure S4. Heat map indicating the relative abundance of minor genera with cumulative abundance of 0.1–0.01%. (PPTX 364 kb) [file 12866_2018_1275_MOESM7_ESM.pptx]

## Slide 1
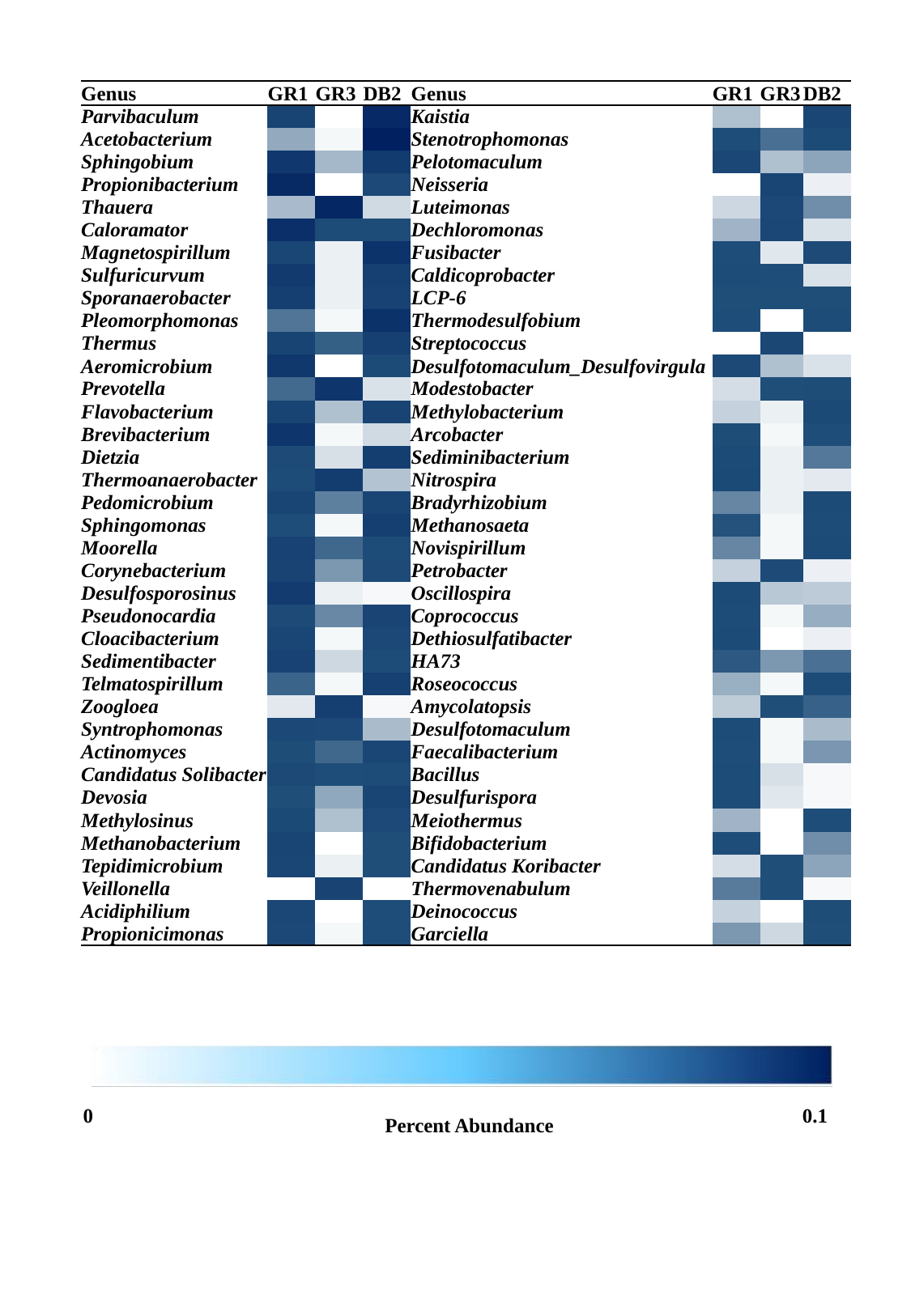

| Genus | GR1 | GR3 | DB2 | Genus | GR1 | GR3 | DB2 |
| --- | --- | --- | --- | --- | --- | --- | --- |
| Parvibaculum | | | | Kaistia | | | |
| Acetobacterium | | | | Stenotrophomonas | | | |
| Sphingobium | | | | Pelotomaculum | | | |
| Propionibacterium | | | | Neisseria | | | |
| Thauera | | | | Luteimonas | | | |
| Caloramator | | | | Dechloromonas | | | |
| Magnetospirillum | | | | Fusibacter | | | |
| Sulfuricurvum | | | | Caldicoprobacter | | | |
| Sporanaerobacter | | | | LCP-6 | | | |
| Pleomorphomonas | | | | Thermodesulfobium | | | |
| Thermus | | | | Streptococcus | | | |
| Aeromicrobium | | | | Desulfotomaculum\_Desulfovirgula | | | |
| Prevotella | | | | Modestobacter | | | |
| Flavobacterium | | | | Methylobacterium | | | |
| Brevibacterium | | | | Arcobacter | | | |
| Dietzia | | | | Sediminibacterium | | | |
| Thermoanaerobacter | | | | Nitrospira | | | |
| Pedomicrobium | | | | Bradyrhizobium | | | |
| Sphingomonas | | | | Methanosaeta | | | |
| Moorella | | | | Novispirillum | | | |
| Corynebacterium | | | | Petrobacter | | | |
| Desulfosporosinus | | | | Oscillospira | | | |
| Pseudonocardia | | | | Coprococcus | | | |
| Cloacibacterium | | | | Dethiosulfatibacter | | | |
| Sedimentibacter | | | | HA73 | | | |
| Telmatospirillum | | | | Roseococcus | | | |
| Zoogloea | | | | Amycolatopsis | | | |
| Syntrophomonas | | | | Desulfotomaculum | | | |
| Actinomyces | | | | Faecalibacterium | | | |
| Candidatus Solibacter | | | | Bacillus | | | |
| Devosia | | | | Desulfurispora | | | |
| Methylosinus | | | | Meiothermus | | | |
| Methanobacterium | | | | Bifidobacterium | | | |
| Tepidimicrobium | | | | Candidatus Koribacter | | | |
| Veillonella | | | | Thermovenabulum | | | |
| Acidiphilium | | | | Deinococcus | | | |
| Propionicimonas | | | | Garciella | | | |
0
0.1
Percent Abundance
