# Supplementary material for: Petroleum hydrocarbon rich oil refinery sludge of North-East India harbours anaerobic, fermentative, sulfate-reducing, syntrophic and methanogenic microbial populations
Source: BMC Microbiol. 2018 Oct 22;18:151. doi: 10.1186/s12866-018-1275-8 (PMC6198496; doi:10.1186/s12866-018-1275-8)
Supplement: Supplementary file 8 — Figure S5. Phylogentic tree representing of clade1 of top 50 most abundant OTUs. Tree was constructed using the neighbour joining method incorporating Jukes-Cantor distance corrections. One thousand bootstrap analyses were conducted and bootstrap values > 50% were indicated at the nodes. Scale bar = 0.05 change per nucleotide position. The values in bracket indicated abundance in following the sequence of GR1/DB2/GR3. (PPTX 91 kb) [file 12866_2018_1275_MOESM8_ESM.pptx]

## Slide 1
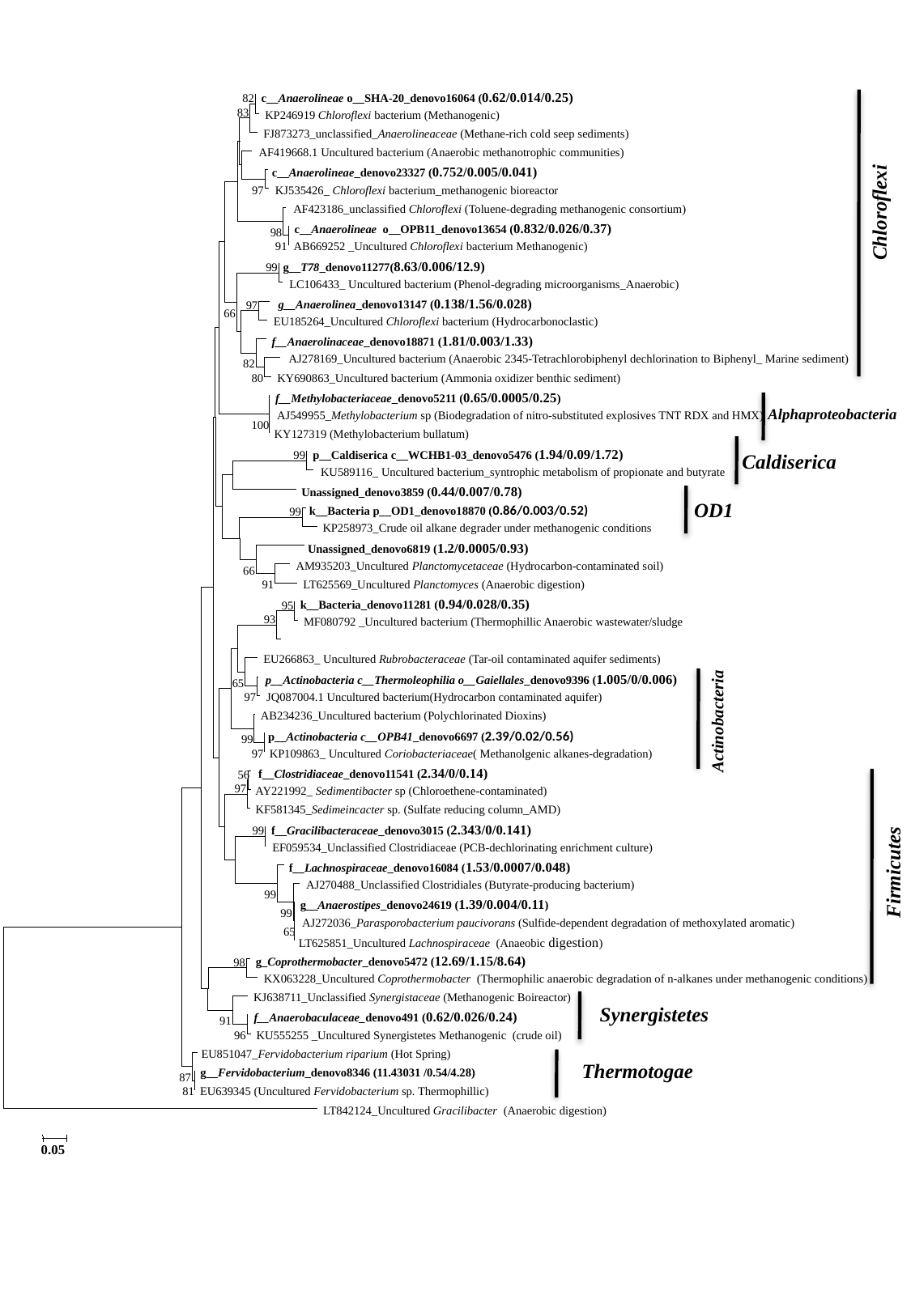

c__Anaerolineae o__SHA-20_denovo16064 (0.62/0.014/0.25)
82
83
 KP246919 Chloroflexi bacterium (Methanogenic)
 FJ873273_unclassified_Anaerolineaceae (Methane-rich cold seep sediments)
 AF419668.1 Uncultured bacterium (Anaerobic methanotrophic communities)
 c__Anaerolineae_denovo23327 (0.752/0.005/0.041)
97
 KJ535426_ Chloroflexi bacterium_methanogenic bioreactor
 AF423186_unclassified Chloroflexi (Toluene-degrading methanogenic consortium)
 c__Anaerolineae o__OPB11_denovo13654 (0.832/0.026/0.37)
98
91
 AB669252 _Uncultured Chloroflexi bacterium Methanogenic)
 g__T78_denovo11277(8.63/0.006/12.9)
99
 LC106433_ Uncultured bacterium (Phenol-degrading microorganisms_Anaerobic)
 g__Anaerolinea_denovo13147 (0.138/1.56/0.028)
97
66
 EU185264_Uncultured Chloroflexi bacterium (Hydrocarbonoclastic)
 f__Anaerolinaceae_denovo18871 (1.81/0.003/1.33)
 AJ278169_Uncultured bacterium (Anaerobic 2345-Tetrachlorobiphenyl dechlorination to Biphenyl_ Marine sediment)
82
80
 KY690863_Uncultured bacterium (Ammonia oxidizer benthic sediment)
 f__Methylobacteriaceae_denovo5211 (0.65/0.0005/0.25)
 AJ549955_Methylobacterium sp (Biodegradation of nitro-substituted explosives TNT RDX and HMX)
100
 KY127319 (Methylobacterium bullatum)
 p__Caldiserica c__WCHB1-03_denovo5476 (1.94/0.09/1.72)
99
 KU589116_ Uncultured bacterium_syntrophic metabolism of propionate and butyrate
 Unassigned_denovo3859 (0.44/0.007/0.78)
 k__Bacteria p__OD1_denovo18870 (0.86/0.003/0.52)
99
 KP258973_Crude oil alkane degrader under methanogenic conditions
 Unassigned_denovo6819 (1.2/0.0005/0.93)
 AM935203_Uncultured Planctomycetaceae (Hydrocarbon-contaminated soil)
66
91
 LT625569_Uncultured Planctomyces (Anaerobic digestion)
 k__Bacteria_denovo11281 (0.94/0.028/0.35)
95
93
 MF080792 _Uncultured bacterium (Thermophillic Anaerobic wastewater/sludge
 EF205582_ Uncultured candidate division OP7 (Thermophillic microbial mats from hot spring ) ermophilic microbial mats from hot springs)
 EU266863_ Uncultured Rubrobacteraceae (Tar-oil contaminated aquifer sediments)
 p__Actinobacteria c__Thermoleophilia o__Gaiellales_denovo9396 (1.005/0/0.006)
65
97
 JQ087004.1 Uncultured bacterium(Hydrocarbon contaminated aquifer)
 AB234236_Uncultured bacterium (Polychlorinated Dioxins)
 p__Actinobacteria c__OPB41_denovo6697 (2.39/0.02/0.56)
99
97
 KP109863_ Uncultured Coriobacteriaceae( Methanolgenic alkanes-degradation)
 f__Clostridiaceae_denovo11541 (2.34/0/0.14)
56
97
 AY221992_ Sedimentibacter sp (Chloroethene-contaminated)
 KF581345_Sedimeincacter sp. (Sulfate reducing column_AMD)
 f__Gracilibacteraceae_denovo3015 (2.343/0/0.141)
99
 EF059534_Unclassified Clostridiaceae (PCB-dechlorinating enrichment culture)
 f__Lachnospiraceae_denovo16084 (1.53/0.0007/0.048)
 AJ270488_Unclassified Clostridiales (Butyrate-producing bacterium)
99
 g__Anaerostipes_denovo24619 (1.39/0.004/0.11)
99
 AJ272036_Parasporobacterium paucivorans (Sulfide-dependent degradation of methoxylated aromatic)
65
 LT625851_Uncultured Lachnospiraceae (Anaeobic digestion)
 g_Coprothermobacter_denovo5472 (12.69/1.15/8.64)
98
 KX063228_Uncultured Coprothermobacter (Thermophilic anaerobic degradation of n-alkanes under methanogenic conditions)
 KJ638711_Unclassified Synergistaceae (Methanogenic Boireactor)
 f__Anaerobaculaceae_denovo491 (0.62/0.026/0.24)
91
96
 KU555255 _Uncultured Synergistetes Methanogenic (crude oil)
 EU851047_Fervidobacterium riparium (Hot Spring)
 g__Fervidobacterium_denovo8346 (11.43031 /0.54/4.28)
87
81
 EU639345 (Uncultured Fervidobacterium sp. Thermophillic)
 LT842124_Uncultured Gracilibacter (Anaerobic digestion)
0.05
Chloroflexi
Alphaproteobacteria
Caldiserica
OD1
Actinobacteria
Firmicutes
Synergistetes
Thermotogae
