# Supplementary material for: Petroleum hydrocarbon rich oil refinery sludge of North-East India harbours anaerobic, fermentative, sulfate-reducing, syntrophic and methanogenic microbial populations
Source: BMC Microbiol. 2018 Oct 22;18:151. doi: 10.1186/s12866-018-1275-8 (PMC6198496; doi:10.1186/s12866-018-1275-8)
Supplement: Supplementary file 9 — Figure S6. Phylogentic tree representing of clade 2 of top 50 most abundant OTUs. Tree was constructed using the neighbour joining method incorporating Jukes-Cantor distance corrections. One thousand bootstrap analyses were conducted and bootstrap values > 50% were indicated at the nodes. Scale bar = 0.02 change per nucleotide position. The values in bracket indicated abundance in following the sequence of GR1/DB2/GR3. (PPTX 74 kb) [file 12866_2018_1275_MOESM9_ESM.pptx]

## Slide 1
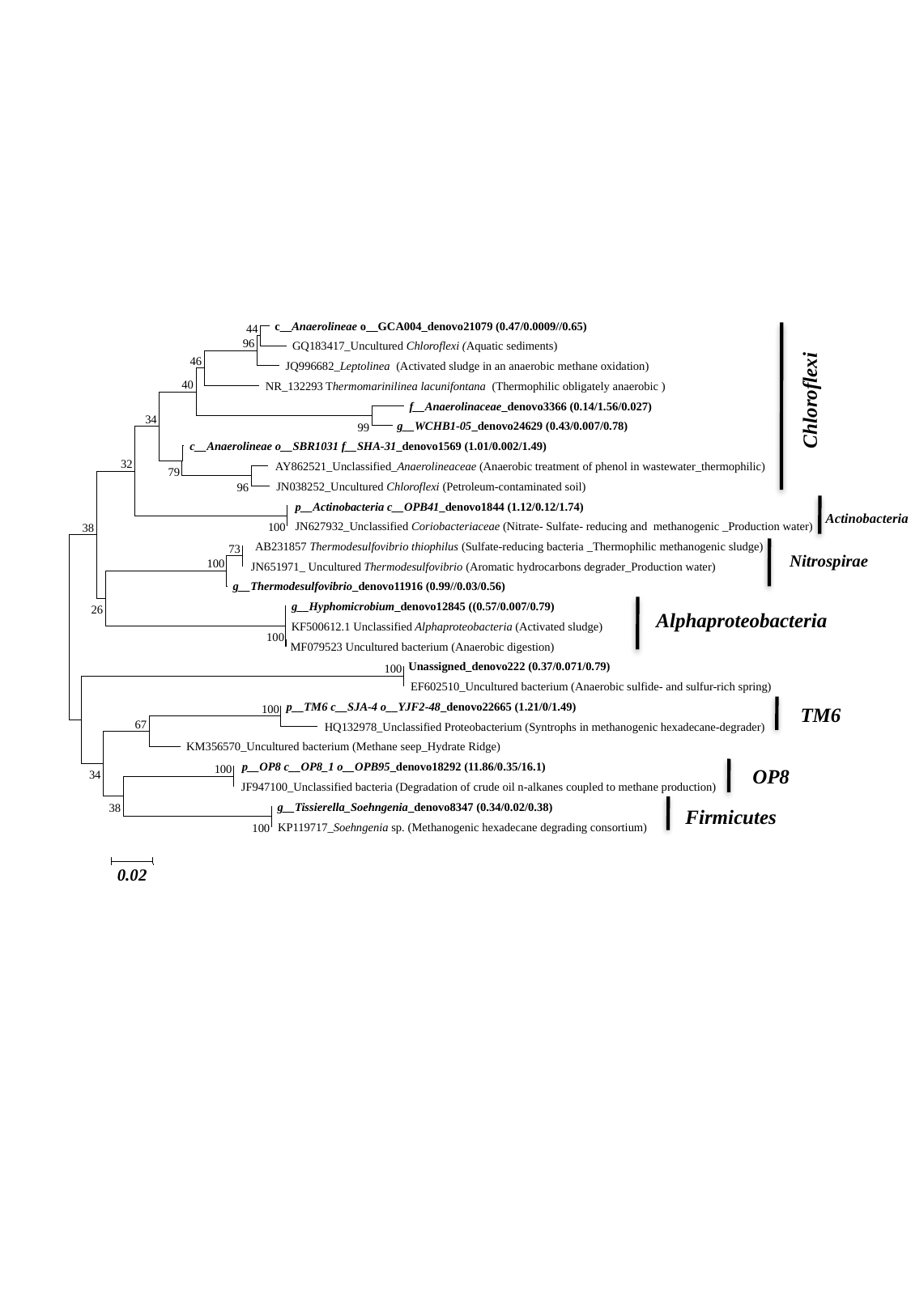

c__Anaerolineae o__GCA004_denovo21079 (0.47/0.0009//0.65)
44
96
 GQ183417_Uncultured Chloroflexi (Aquatic sediments)
46
 JQ996682_Leptolinea (Activated sludge in an anaerobic methane oxidation)
40
 NR_132293 Thermomarinilinea lacunifontana (Thermophilic obligately anaerobic )
 f__Anaerolinaceae_denovo3366 (0.14/1.56/0.027)
34
 g__WCHB1-05_denovo24629 (0.43/0.007/0.78)
99
 c__Anaerolineae o__SBR1031 f__SHA-31_denovo1569 (1.01/0.002/1.49)
32
 AY862521_Unclassified_Anaerolineaceae (Anaerobic treatment of phenol in wastewater_thermophilic)
79
 JN038252_Uncultured Chloroflexi (Petroleum-contaminated soil)
96
 p__Actinobacteria c__OPB41_denovo1844 (1.12/0.12/1.74)
 JN627932_Unclassified Coriobacteriaceae (Nitrate- Sulfate- reducing and methanogenic _Production water)
100
38
 AB231857 Thermodesulfovibrio thiophilus (Sulfate-reducing bacteria _Thermophilic methanogenic sludge)
73
100
 JN651971_ Uncultured Thermodesulfovibrio (Aromatic hydrocarbons degrader_Production water)
 g__Thermodesulfovibrio_denovo11916 (0.99//0.03/0.56)
 g__Hyphomicrobium_denovo12845 ((0.57/0.007/0.79)
26
 KF500612.1 Unclassified Alphaproteobacteria (Activated sludge)
100
 MF079523 Uncultured bacterium (Anaerobic digestion)
 Unassigned_denovo222 (0.37/0.071/0.79)
100
 EF602510_Uncultured bacterium (Anaerobic sulfide- and sulfur-rich spring)
 p__TM6 c__SJA-4 o__YJF2-48_denovo22665 (1.21/0/1.49)
100
67
 HQ132978_Unclassified Proteobacterium (Syntrophs in methanogenic hexadecane-degrader)
 KM356570_Uncultured bacterium (Methane seep_Hydrate Ridge)
 p__OP8 c__OP8_1 o__OPB95_denovo18292 (11.86/0.35/16.1)
100
34
 JF947100_Unclassified bacteria (Degradation of crude oil n-alkanes coupled to methane production)
 g__Tissierella_Soehngenia_denovo8347 (0.34/0.02/0.38)
38
 KP119717_Soehngenia sp. (Methanogenic hexadecane degrading consortium)
100
0.02
Chloroflexi
Actinobacteria
Nitrospirae
Alphaproteobacteria
TM6
OP8
Firmicutes
