# Supplementary material for: Petroleum hydrocarbon rich oil refinery sludge of North-East India harbours anaerobic, fermentative, sulfate-reducing, syntrophic and methanogenic microbial populations
Source: BMC Microbiol. 2018 Oct 22;18:151. doi: 10.1186/s12866-018-1275-8 (PMC6198496; doi:10.1186/s12866-018-1275-8)
Supplement: Supplementary file 10 — Figure S7. Phylogentic tree representing of clade 3 of top 50 most abundant OTUs. Tree was constructed using the neighbour joining method incorporating Jukes-Cantor distance corrections. One thousand bootstrap analyses were conducted and bootstrap values > 50% were indicated at the nodes. Scale bar = 0.02 change per nucleotide position. The values in bracket indicated abundance in following the sequence of GR1/DB2/GR3. (PPTX 74 kb) [file 12866_2018_1275_MOESM10_ESM.pptx]

## Slide 1
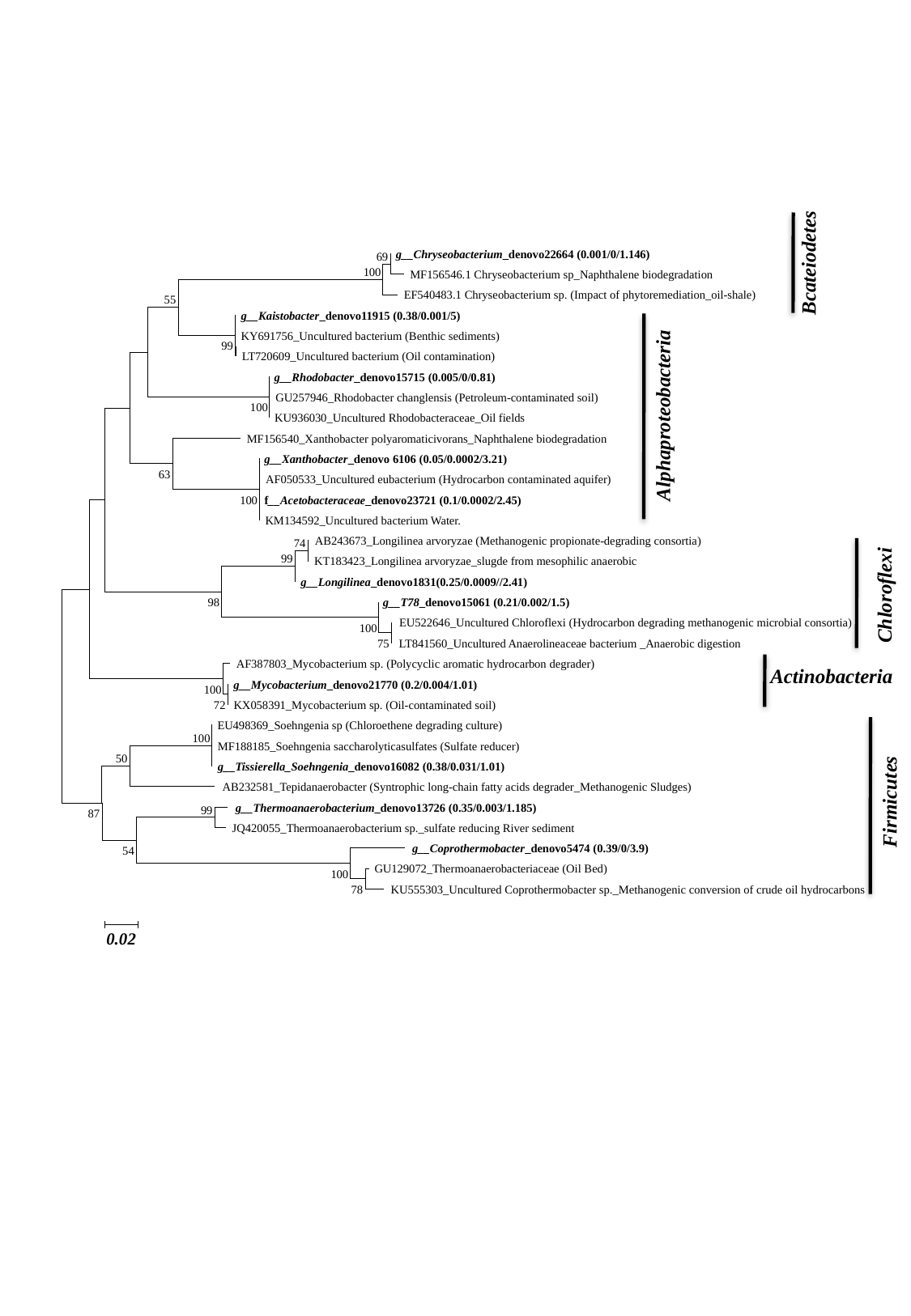

g__Chryseobacterium_denovo22664 (0.001/0/1.146)
69
100
 MF156546.1 Chryseobacterium sp_Naphthalene biodegradation
 EF540483.1 Chryseobacterium sp. (Impact of phytoremediation_oil-shale)
55
 g__Kaistobacter_denovo11915 (0.38/0.001/5)
 KY691756_Uncultured bacterium (Benthic sediments)
99
 LT720609_Uncultured bacterium (Oil contamination)
 g__Rhodobacter_denovo15715 (0.005/0/0.81)
 GU257946_Rhodobacter changlensis (Petroleum-contaminated soil)
100
 KU936030_Uncultured Rhodobacteraceae_Oil fields
 MF156540_Xanthobacter polyaromaticivorans_Naphthalene biodegradation
 g__Xanthobacter_denovo 6106 (0.05/0.0002/3.21)
63
 AF050533_Uncultured eubacterium (Hydrocarbon contaminated aquifer)
100
 f__Acetobacteraceae_denovo23721 (0.1/0.0002/2.45)
 KM134592_Uncultured bacterium Water.
 AB243673_Longilinea arvoryzae (Methanogenic propionate-degrading consortia)
74
99
 KT183423_Longilinea arvoryzae_slugde from mesophilic anaerobic
 g__Longilinea_denovo1831(0.25/0.0009//2.41)
98
 g__T78_denovo15061 (0.21/0.002/1.5)
 EU522646_Uncultured Chloroflexi (Hydrocarbon degrading methanogenic microbial consortia)
100
75
 LT841560_Uncultured Anaerolineaceae bacterium _Anaerobic digestion
 AF387803_Mycobacterium sp. (Polycyclic aromatic hydrocarbon degrader)
 g__Mycobacterium_denovo21770 (0.2/0.004/1.01)
100
72
 KX058391_Mycobacterium sp. (Oil-contaminated soil)
 EU498369_Soehngenia sp (Chloroethene degrading culture)
100
 MF188185_Soehngenia saccharolyticasulfates (Sulfate reducer)
50
 g__Tissierella_Soehngenia_denovo16082 (0.38/0.031/1.01)
 AB232581_Tepidanaerobacter (Syntrophic long-chain fatty acids degrader_Methanogenic Sludges)
 g__Thermoanaerobacterium_denovo13726 (0.35/0.003/1.185)
99
87
 JQ420055_Thermoanaerobacterium sp._sulfate reducing River sediment
 g__Coprothermobacter_denovo5474 (0.39/0/3.9)
54
 GU129072_Thermoanaerobacteriaceae (Oil Bed)
100
78
 KU555303_Uncultured Coprothermobacter sp._Methanogenic conversion of crude oil hydrocarbons
0.02
Bcateiodetes
Alphaproteobacteria
Chloroflexi
Actinobacteria
Firmicutes
