# Supplementary material for: Petroleum hydrocarbon rich oil refinery sludge of North-East India harbours anaerobic, fermentative, sulfate-reducing, syntrophic and methanogenic microbial populations
Source: BMC Microbiol. 2018 Oct 22;18:151. doi: 10.1186/s12866-018-1275-8 (PMC6198496; doi:10.1186/s12866-018-1275-8)
Supplement: Supplementary file 11 — Figure S8. Phylogentic tree representing of clade 4 of top 50 most abundant OTUs. Tree was constructed using the neighbour joining method incorporating Jukes-Cantor distance corrections. One thousand bootstrap analyses were conducted and bootstrap values > 50% were indicated at the nodes. Scale bar = 0.05 change per nucleotide position. The values in bracket indicated abundance in following the sequence of GR1/DB2/GR3. (PPTX 54 kb) [file 12866_2018_1275_MOESM11_ESM.pptx]

## Slide 1
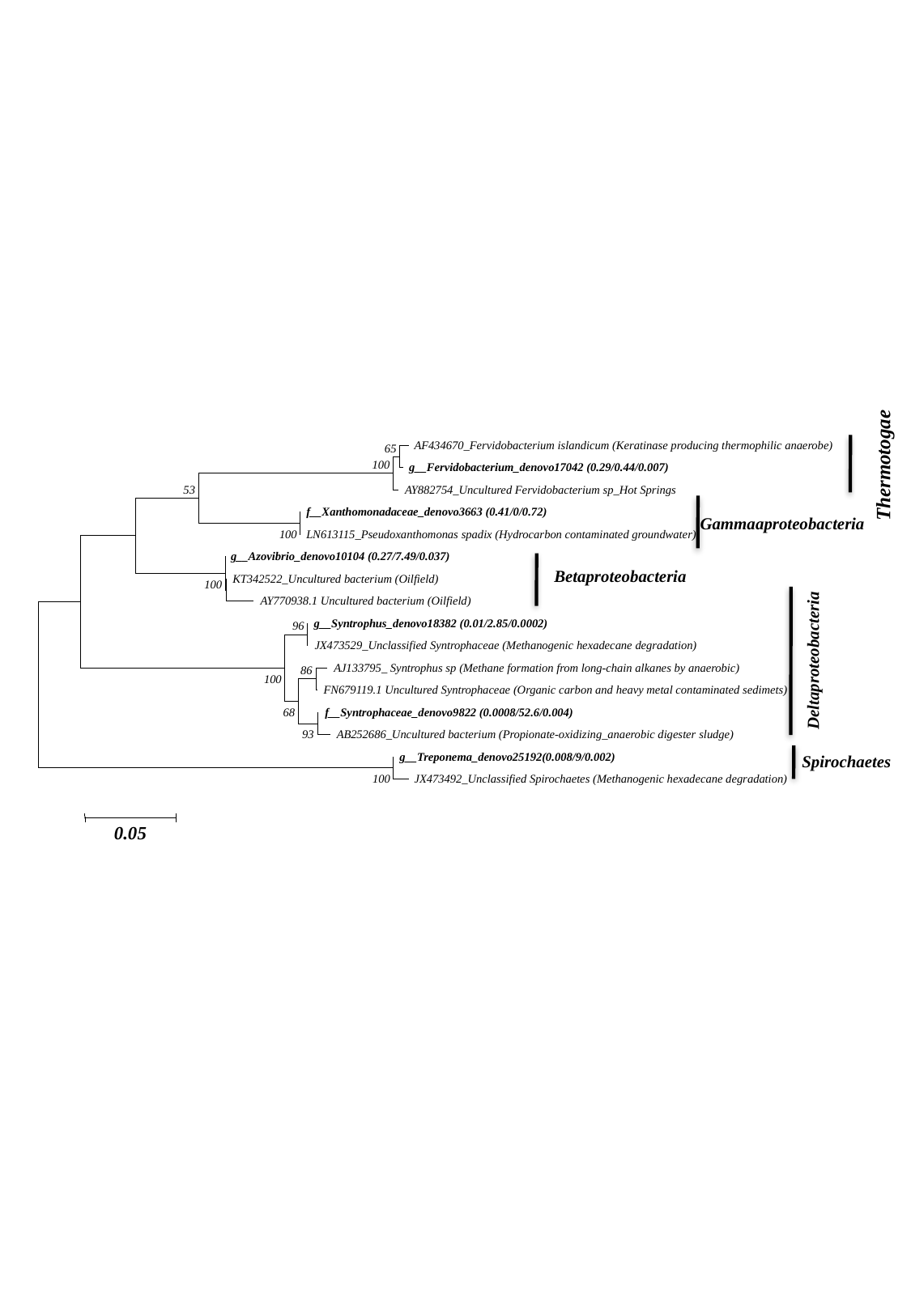

AF434670_Fervidobacterium islandicum (Keratinase producing thermophilic anaerobe)
65
100
 g__Fervidobacterium_denovo17042 (0.29/0.44/0.007)
53
 AY882754_Uncultured Fervidobacterium sp_Hot Springs
 f__Xanthomonadaceae_denovo3663 (0.41/0/0.72)
 LN613115_Pseudoxanthomonas spadix (Hydrocarbon contaminated groundwater)
100
 g__Azovibrio_denovo10104 (0.27/7.49/0.037)
 KT342522_Uncultured bacterium (Oilfield)
100
 AY770938.1 Uncultured bacterium (Oilfield)
 g__Syntrophus_denovo18382 (0.01/2.85/0.0002)
96
 JX473529_Unclassified Syntrophaceae (Methanogenic hexadecane degradation)
 AJ133795_ Syntrophus sp (Methane formation from long-chain alkanes by anaerobic)
86
100
 FN679119.1 Uncultured Syntrophaceae (Organic carbon and heavy metal contaminated sedimets)
 f__Syntrophaceae_denovo9822 (0.0008/52.6/0.004)
68
 AB252686_Uncultured bacterium (Propionate-oxidizing_anaerobic digester sludge)
93
 g__Treponema_denovo25192(0.008/9/0.002)
 JX473492_Unclassified Spirochaetes (Methanogenic hexadecane degradation)
100
0.05
Thermotogae
Gammaaproteobacteria
Betaproteobacteria
Deltaproteobacteria
Spirochaetes
