# Supplementary material for: Petroleum hydrocarbon rich oil refinery sludge of North-East India harbours anaerobic, fermentative, sulfate-reducing, syntrophic and methanogenic microbial populations
Source: BMC Microbiol. 2018 Oct 22;18:151. doi: 10.1186/s12866-018-1275-8 (PMC6198496; doi:10.1186/s12866-018-1275-8)
Supplement: Supplementary file 12 — Table S4. Details of different hydrocarbon contaminated samples considered for comparative analysis. (DOCX 12 kb) [file 12866_2018_1275_MOESM12_ESM.docx]

**Table S4. Details of different hydrocarbon contaminated samples considered for comparative analysis**

| **Name of the Sample** | **Nature of the Sample** | **Reference** |
| --- | --- | --- |
| GR1 | Test Sample |  |
| GR3 | Test Sample |  |
| DB2 | Test Sample |  |
| A3 | Oil contaminated sediments | Gao et al., 2018 |
| B3 | Oil contaminated sediments | Gao et al., 2018 |
| C3 | Oil contaminated sediments | Gao et al., 2018 |
| NA | Natural attenuated oil contaminated soil | Wu et al., 2017 |
| CT | Control oil contaminated soil | Wu et al., 2017 |
| BR | Bioremediated oil contaminated soil | Wu et al., 2017 |
| DQ | Daqing (DQ) oil field northeast China | Liao et al., 2015 |
| XJ | Karamay (XJ) oil field in western China, | Liao et al., 2015 |
| SL | Shengli (SL) oil field Yellow River area in northern China | Liao et al., 2015 |
| HB | Huabei (HB) oil field Huabei plain, northern China | Liao et al., 2015 |
| B3_RS | Refuelling station soil | Sutton et al., 2013 |
| B4_RS | Refuelling station soil | Sutton et al., 2013 |
| A2_RS | Refuelling station soil | Sutton et al., 2013 |
| JP-8 fuel | JP-8 fuel spill Arctic soil | Bell et al 2011 |
| SU3 | Tailings pond | Yergeau et al., 2012 |
