# Supplementary material for: Petroleum hydrocarbon rich oil refinery sludge of North-East India harbours anaerobic, fermentative, sulfate-reducing, syntrophic and methanogenic microbial populations
Source: BMC Microbiol. 2018 Oct 22;18:151. doi: 10.1186/s12866-018-1275-8 (PMC6198496; doi:10.1186/s12866-018-1275-8)
Supplement: Supplementary file 13 — Table S5. Detailed distribution of predictive genes within sludge metagenomes using PICRUSt. (DOC 87 kb) [file 12866_2018_1275_MOESM13_ESM.doc]

**Table S4 Detailed distribution of predictive genes within sludge metagenomes using PICRUSt**

| **KEGG_Pathways** | **GR1** | **GR3** | **DB2** |
| --- | --- | --- | --- |
| ^b^Pyruvate metabolism | 1.21977 | 1.12044 | 1.23876 |
| ^a^Citrate cycle (TCA cycle) | 1.0848 | 0.83251 | 1.05539 |
| ^a^Galactose metabolism | 0.58149 | 0.28667 | 0.55493 |
| ^a^Glycolysis / Gluconeogenesis | 1.31912 | 0.96188 | 1.29598 |
| ^c^Butanoate metabolism | 0.84991 | 1.05172 | 0.96207 |
| ^b^Propanoate metabolism | 0.87455 | 0.91511 | 0.967 |
| ^c^Glyoxylate and dicarboxylate metabolism | 0.535 | 0.68689 | 0.6298 |
| ^a^Other Carbohydrate Metabolism | 4.5956 | 3.35339 | 4.4995 |
| ^a^Amino acid related enzymes | 1.40534 | 1.35378 | 1.33 |
| ^b^Arginine and proline metabolism | 1.24495 | 0.92904 | 1.2703 |
| ^b^Cysteine and methionine metabolism | 0.92066 | 0.741086 | 0.869856 |
| ^b^Other Amino Acid Metabolism | 6.67511 | 6.22118 | 7.14202 |
| ^a^Carbon fixation in photosynthetic organisms | 0.61016 | 0.48899 | 0.58668 |
| ^c^Carbon fixation pathways in prokaryotes | 1.35466 | 1.35941 | 1.28221 |
| ^c^Methane metabolism | 1.24612 | 1.29441 | 1.2188 |
| ^c^Nitrogen metabolism | 0.62638 | 0.92514 | 0.63867 |
| ^b^Oxidative phosphorylation | 1.55497 | 1.16007 | 1.57712 |
| ^b^Photosynthesis | 0.36119 | 0.23963 | 0.32816 |
| ^b^Photosynthesis - antenna proteins | 0.00023 | 0.00013 | 0.0003 |
| ^a^Photosynthesis proteins | 0.37995 | 0.26319 | 0.34745 |
| ^c^Sulfur metabolism | 0.22797 | 0.25943 | 0.25585 |
| ^c^1,1,1-Trichloro-2,2-bis(4-chlorophenyl)ethane (DDT) degradation | 0.000692 | 0.009674 | 0.000836 |
| ^b^Aminobenzoate degradation | 0.309238 | 0.320964 | 0.405343 |
| ^b^Atrazine degradation | 0.042668 | 0.01749 | 0.064434 |
| ^c^Benzoate degradation | 0.290885 | 0.554595 | 0.431151 |
| ^b^Bisphenol degradation | 0.104939 | 0.073291 | 0.136175 |
| ^c^Caprolactam degradation | 0.127226 | 0.20262 | 0.197812 |
| ^b^Chloroalkane and chloroalkene degradation | 0.191813 | 0.227247 | 0.2478 |
| ^b^Chlorocyclohexane and chlorobenzene degradation | 0.040562 | 0.067601 | 0.075623 |
| ^c^Dioxin degradation | 0.044575 | 0.087769 | 0.059148 |
| ^b^Drug metabolism - cytochrome P450 | 0.088694 | 0.085746 | 0.150469 |
| ^a^Drug metabolism - other enzymes | 0.282529 | 0.206797 | 0.265659 |
| ^c^Ethylbenzene degradation | 0.036049 | 0.074614 | 0.049319 |
| ^b^Fluorobenzoate degradation | 0.020606 | 0.015372 | 0.044776 |
| ^b^Metabolism of xenobiotics by cytochrome P450 | 0.08221 | 0.083589 | 0.142124 |
| ^c^Naphthalene degradation | 0.143379 | 0.200624 | 0.198267 |
| ^c^Nitrotoluene degradation | 0.236215 | 0.274164 | 0.205234 |
| ^b^Polycyclic aromatic hydrocarbon degradation | 0.114759 | 0.102734 | 0.15274 |
| ^b^Styrene degradation | 0.058062 | 0.069318 | 0.083945 |
| ^b^Toluene degradation | 0.128035 | 0.122725 | 0.167198 |
| ^c^Xylene degradation | 0.048598 | 0.085202 | 0.057022 |
| ^b^Lipid biosynthesis proteins | 0.795149 | 0.801303 | 0.851225 |
| ^c^Fatty acid biosynthesis | 0.59142 | 0.457625 | 0.59396 |
| ^b^Fatty acid metabolism | 0.47133 | 0.68516 | 0.63338 |
| ^b^Other Lipid Metabolism | 1.635732 | 1.263521 | 1.758387 |
| ^a^Metabolism of Cofactors and Vitamins | 4.46337 | 4.17884 | 4.35572 |
| ^b^Metabolism of Terpenoids and Polyketides | 1.97697 | 1.9169 | 2.13571 |
| ^b^Metabolism of Other Amino Acids | 1.52832 | 1.53226 | 1.69432 |
| ^a^Glycan Biosynthesis and Metabolism | 2.0186 | 2.0176 | 1.98697 |
| ^a^Cytochrome P450 | 0.00062 | 7.90E-05 | 0.00118 |
| ^b^Peptidases | 1.5203 | 1.28286 | 1.45517 |
| ^c^Protein kinases | 0.27967 | 0.46024 | 0.27791 |
| ^b^Biosynthesis of Other Secondary Metabolites | 1.05489 | 0.76415 | 1.0927 |
| ^a^Purine metabolism | 2.17129 | 1.8327 | 2.04462 |
| ^a^Pyrimidine metabolism | 1.69643 | 1.4489 | 1.49773 |
| ^b^ABC transporters | 3.83019 | 3.6451 | 3.98644 |
| ^b^Transporters | 6.50726 | 5.55345 | 6.77195 |
| ^c^Two-component system | 1.52415 | 2.75985 | 1.55372 |
| ^c^Secretion system | 1.48994 | 2.03976 | 1.37927 |
| ^c^Bacterial secretion system | 0.62107 | 0.67517 | 0.63769 |
| ^a^Bacterial toxins | 0.08279 | 0.0658 | 0.0764 |
| ^a^Other Environmental Information Processing | 0.3291 | 0.29061 | 0.2939 |
| ^c^Bacterial motility proteins | 1.60305 | 2.84302 | 1.29364 |
| ^c^Flagellar assembly | 0.75006 | 1.19232 | 0.56639 |
| ^c^Other Cellular Processes | 1.64437 | 2.36702 | 1.60518 |
| ^a^DNA repair and recombination proteins | 2.464941 | 2.390645 | 2.341241 |
| ^a^Ribosome | 2.326324 | 1.884693 | 2.080945 |
| ^a^Aminoacyl-tRNA biosynthesis | 1.283705 | 1.088374 | 1.171194 |
| ^a^Transcription factors | 1.247637 | 1.131015 | 1.218285 |
| ^c^Other Genetic Information | 10.2464 | 9.94692 | 9.53782 |
| ^b^Human Diseases | 0.68858 | 0.92114 | 0.85112 |
| ^c^Organismal Systems | 0.67978 | 0.8681 | 0.72217 |
| ^c^Unclassified | 12.417 | 14.3686 | 12.3477 |

^a^ maximum in GR1, ^b^ maximum in DB2 and ^c^ maximum in GR3
